# Microbiome-based classification models for fresh produce safety and quality evaluation

**DOI:** 10.1128/spectrum.03448-23

**Published:** 2024-03-06

**Authors:** Chao Liao, Luxin Wang, Gerald Quon

**Affiliations:** 1 Department of Food Science and Technology, University of California Davis, Davis, California, USA; 2 Department of Molecular and Cellular Biology, University of California Davis, Davis, California, USA; University of Torino, Torino, Italy

**Keywords:** produce safety, produce quality, machine learning, random forest, k-mer hash, amplicon sequence variant

## Abstract

Small sample sizes and loss of sequencing reads during the microbiome data preprocessing can limit the statistical power of differentiating fresh produce phenotypes and prevent the detection of important bacterial species associated with produce contamination or quality reduction. Here, we explored a machine learning-based *k*-mer hash analysis strategy to identify DNA signatures predictive of produce safety (PS) and produce quality (PQ) and compared it against the amplicon sequence variant (ASV) strategy that uses a typical denoising step and ASV-based taxonomy strategy. Random forest-based classifiers for PS and PQ using 7-mer hash data sets had significantly higher classification accuracy than those using the ASV data sets. We also demonstrated that the proposed combination of integrating multiple data sets and leveraging a 7-mer hash strategy leads to better classification performance for PS and PQ compared to the ASV method but presents lower PS classification accuracy compared to the feature-selected ASV-based taxonomy strategy. Due to the current limitation of generating taxonomy using the 7-mer hash strategy, the ASV-based taxonomy strategy with remarkably less computing time and memory usage is more efficient for PS and PQ classification and applicable for important taxa identification. Results generated from this study lay the foundation for future studies that wish and need to incorporate and/or compare different microbiome sequencing data sets for the application of machine learning in the area of microbial safety and quality of food.

**IMPORTANCE:**

Identification of generalizable indicators for produce safety (PS) and produce quality (PQ) improves the detection of produce contamination and quality decline. However, effective sequencing read loss during microbiome data preprocessing and the limited sample size of individual studies restrain statistical power to identify important features contributing to differentiating PS and PQ phenotypes. We applied machine learning-based models using individual and integrated *k*-mer hash and amplicon sequence variant (ASV) data sets for PS and PQ classification and evaluated their classification performance and found that random forest (RF)-based models using integrated 7-mer hash data sets achieved significantly higher PS and PQ classification accuracy. Due to the limitation of taxonomic analysis for the 7-mer hash, we also developed RF-based models using feature-selected ASV-based taxonomic data sets, which performed better PS classification than those using the integrated 7-mer hash data set. The RF feature selection method identified 480 PS indicators and 263 PQ indicators with a positive contribution to the PS and PQ classification.

## INTRODUCTION

Next-generation sequencing approaches for the analysis of microbial communities in fresh produce include amplicon-based sequencing (e.g., 16S rRNA gene sequencing) and metagenomic sequencing (e.g., shotgun sequencing). These technologies help identify microbial populations within individual produce samples, traditionally by aligning sequenced reads to known genomes to quantify the presence of known species in a sample (1). When multiple samples are sequenced, statistical and machine learning (ML) approaches can be leveraged to identify microbes of importance to produce safety (PS) and produce quality (PQ) (2–12). PS and PQ are the food safety and quality of produce. Generally, food safety has been defined as “the biological, chemical, or physical status of a food that will permit its consumption without incurring excessive risk of injury, morbidity, or mortality” (13). Food quality represents the sum of all properties and assessable attributes, including sensory value, suitability value, and health value, of a food item (14). In this study, PS and PQ refer to microbial food safety and quality. Interactions between pathogenic and/or spoilage microorganisms and other native or background microbiota shine a light on the identification of potential antagonistic microorganisms from produce microbiota to protect and improve the safety and quality of fresh produce (9, 11, 12, 15, 16).

Alignment-free *k*-mer hash-based ML technique has emerged in various metagenome studies. Rowe et al. (17) trained and tested a random forest (RF) classifier using a *k*-mer hash data set containing 108 novel metagenomic microbiome samples from a cohort of premature neonates (17). The classifier predicted whether a neonate was treated with antibiotics in 97% accuracy. Johnson et al. (18) constructed RF models with metagenomic data sets in the *k*-mer format from healthy and pathogen-infected plants, which performed with an accuracy over 90% to detect plant diseases (18). However, limited information has been reported on the application of *k*-mer hash-based ML models in amplicon sequencing microbiome studies.

Of particular interest in this study is the use of ML to identify indicators: bacterial species whose presence is correlated with PS or PQ. Our previous study (11) has applied the linear discriminant analysis effect size method to identify *Arthrobacter*, *Shewanella*, *Brochothrix*, *Rhizobium*, *Novosphingobium*, *Lactococcus*, *Ochrobactrum*, *Variovorax*, *Dyadobacter*, *Methylotenera*, *Yersinia*, and *Wautersiella* as indicators for *Escherichia coli* O157:H7 contamination of spring mix salad. Indicators can be identified by constructing classification models that identify produce sample features that distinguish samples of different PQ or PS statuses. Both the accuracy of the classifiers and the quality and reproducibility of the indicators identified from them typically increase with data set and sample sizes (19). When reviewing and processing published produce microbiome data sets, we found no overlap of PS-related indicators across three PS-related studies (11, 12, 20) and only one indicator, *Leuconostoc*, was observed in two (9, 20) out of three PQ-related studies (9, 11, 20) as illustrated in Fig. S6 and S7. This poor overlap of indicators suggests an opportunity to explore alternative indicator identification strategies that identify more reproducible indicators across studies.

The poor overlap of indicators identified by different studies may be driven by two reasons. The first reason is due to the limited sample sizes in each individual study. On average, 110 samples are analyzed in previously published studies (1, 4–12, 21), which is much smaller than that suggested given the large number of microbial species being identified in each sample (22). Small sample sizes coupled with the profiling of much more microbial species can lead to more spurious (false) indicators or correlations between bacterial species occurrence and pathogen contamination or quality decline and ultimately yield poor reproducibility between studies (11, 12, 15). Secondly, this poor overlap might be caused by low effective reads of each produce sample. For the 16S rRNA gene sequencing data analysis, in addition to the sequencing depth limitations, a large number of reads can be lost through the denoising step in the microbiome data analysis. During the denoising process, up to 50% of reads for the 16S rRNA gene sequencing can be removed due to their low-quality or noising sequences (23, 24). This loss of nearly half of the sequencing reads may reduce the power to identify low abundant species or sequence variations (25); we hypothesize that some of these reads still contain distinguishing information for contributing to the classification of sample phenotypes.

**Fig 1:**
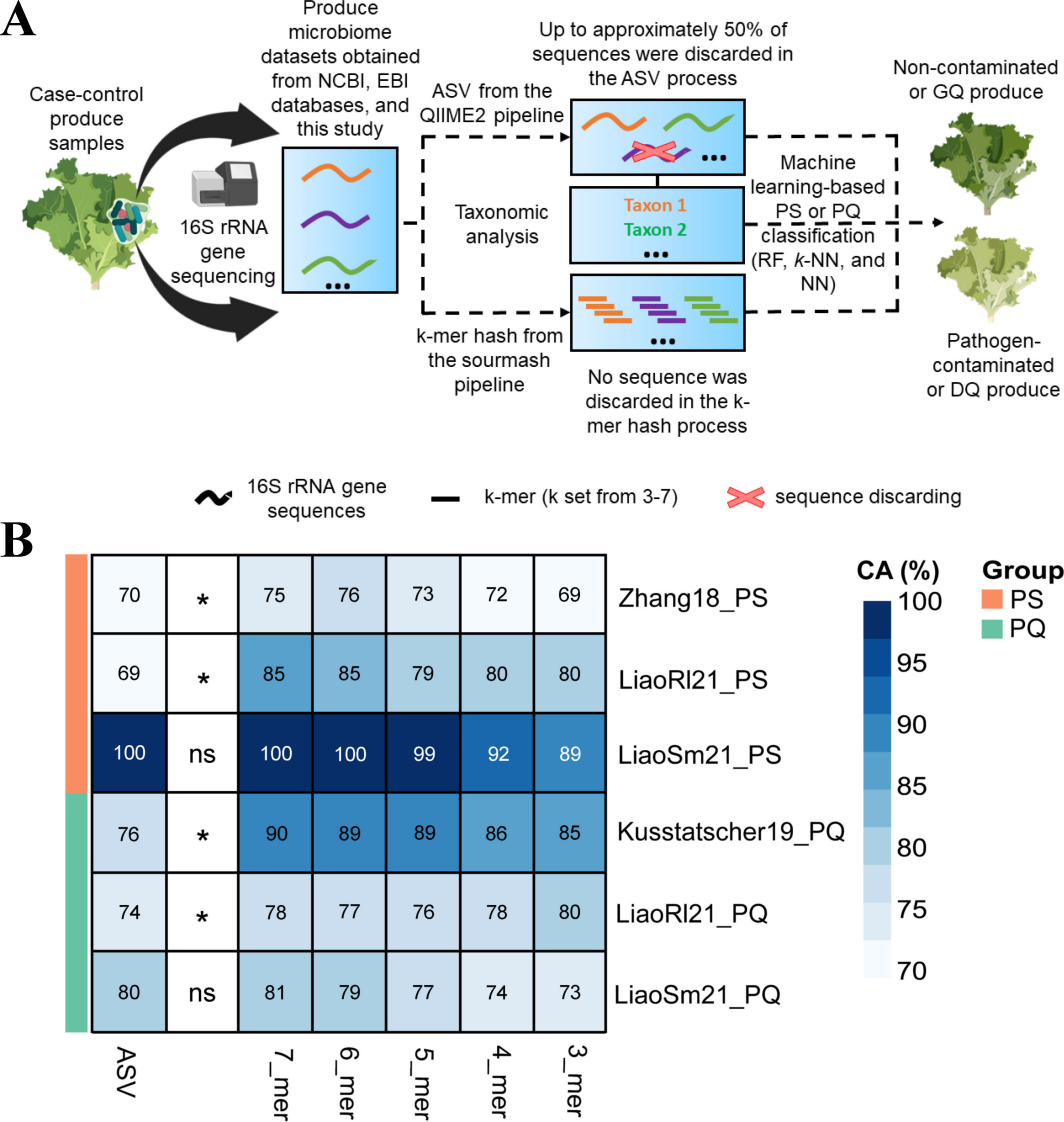
Construction and evaluation of ML-based PS and PQ classifiers. (**A**) Schematic comparison of the preprocessing including denoising of the ASV approach and ASV approach and ASV-based taxonomy approach for constructing IPS and IPQ based on RF in the following section against the preprocessing of the *k*-mer hash approach for constructing PS and PQ classifiers. (**B**) Heatmap of the accuracies achieved by RF-based models using fresh produce microbiome data sets associated with PS and PQ in ASV format and *k*-mer hash format (*k* from 3 to 7). Accuracies are measured using 10-fold cross-validation. GQ and DQ represent good-quality produce and decreasing-quality produce, respectively. CA is classification accuracy. Partial icons in **A** were obtained from “BioRender.com.” Non-parametric Wilcoxon rank sum test was applied to analyze the difference in classification accuracy between ASV-based models and 7-mer hash-based models. The * represents *P* < 0.05 obtained in the statistical test indicating that a statistically significant difference was detected between the two models.

Here, we proposed a computational strategy to address both challenges in order to better identify bacterial indicators broadly correlated with both PS and PQ phenotypes. First, we used an approach based on counting short *k*-mers in sequencing reads (26, 27) to classify fresh produce samples based on detecting the differences in microbial DNA sequences between distinct PS or PQ statuses (Fig. 1A). We showed that *k*-mer hash-based models were significantly better at predicting both PS and PQ compared to the commonly used approach of counting amplicon sequence variants (ASVs) but had lower PS classification accuracy than feature-selected ASV-based taxonomy models. Second, we integrated PS and PQ data sets before analysis to boost data set size and power, and by doing so, we are able to identify taxa that are more broadly associated with PS and PQ.

## MATERIALS AND METHODS

### Fresh produce microbiome data sets

Four published fresh produce microbiome studies that generated 16S rRNA gene sequencing data were selected, as they all have complete metadata information and are associated with PS and/or PQ. These four studies are named Zhang18 (12) (*n* = 236), Kusstatscher19 (9) (*n* = 227), LiaoSm21 (11) (*n* = 72), and LiaoRl21P (20) (*n* = 36). (Table 1). The published non-contaminated samples (LiaoRl21P) (20) and unpublished data from pathogen-contaminated samples (LiaoRl21U, *n* = 72) prepared and sequenced for this paper were combined as one LiaoRl21 data set for use. The preparation of the contaminated samples, DNA extraction step, and library preparation and sequencing followed the protocol published by Liao and Wang (11) and addressed in the additional experimental details. The new data have also been uploaded to the National Center for Biotechnology Information database, and the accession number is PRJNA792031. Based on the three PS-related studies, we created the PS data set composed of case-control studies in which fresh produce was artificially inoculated with *E. coli* O157:H7 (LiaoSm21 and LiaoRl21), *Listeria monocytogenes* (LiaoRl21), or *Salmonella* Infantis (Zhang18). Produce samples without contamination of pathogens were labeled as non-contaminated, while all other samples were given the uniform label contaminated. Also, from the three PQ-related studies, we created the PQ data set in which samples were either labeled as good-quality (GQ samples sequenced before their use by dates or showing no decaying signs) or decreasing-quality (DQ samples after their use by date or decayed) (LiaoSm21, LiaoRl21, and Kusstatscher19).

**TABLE 1:** Characteristics of fresh produce microbiome data sets^*a*^

Data set	Produce types	Sequencing instrument	Sequencing region	Factors	Sample size	References	Database (accession number)
1	Lettuce	Illumina MiSeq	515F/806R (V4)	Contamination treatment Sample type Soil texture Cultivar Harvest time	236	Zhang et al. (12)	NCBI (PRJNA289142)
2	Spring mix salad	Illumina MiSeq	314F/785R (V3 and V4)	Contamination treatment Quality Brand	72	Liao and Wang (11)	EMBL-EBI (ERP112563)
3	Romaine lettuce	Illumina MiSeq	314F/785R (V3 and V4)	Contamination treatment Quality Brand Season Farming practice	108	This study	NCBI (PRJNA792031)
4	Sugar beet	Illumina MiSeq	515F/926R (V4 and V5)	Quality Location Clamp type Amplicon type	227	Kusstatscher et al. (9)	NCBI (PRJEB28964)

^*a*^ EMBL-EBI, European Molecular Biology Laboratory’s European Bioinformatics Institute; NCBI, National Center for Biotechnology Information.

### ASV data set and *k*-mer hash data set preparation

For each data set, raw 16S rRNA gene sequences were imported into the QIIME 2 (version 2021.8) pipeline (28) by using the “qiime tools import” command. The barcodes and primers were removed by using “qiime cutadapt trim-paired/single” plugin of cutadapt (29). Bases in reads with median Phred quality scores of less than 30 were removed by truncating reads with a certain length using the “qiime dada2 denoise-paired/single” plugin of DADA2 (30, 31). During this process, raw sequences were filtered, denoised, chimera-removed, dereplicated, and clustered into ASV (32). Sequences with barcode and primer sequences that were removed were then processed for quality control by truncating bases with the median Phred quality scores of less than 30 with the DADA2 package in R without ASV clustering (30, 31). After that, the processed sequences were used to compute *k*-mer hash signatures (*k* = 3, …, 7) for each sample by using the “sourmash sketch dna” command in the sourmash pipeline (version 4.2.3) (26).

### Common sum scaling

As the sequencing depth varies across samples, data sets, and studies as shown in Fig. S1, the common sum scaling (COM) method was applied to normalize the sequencing depth among them for both ASV and *k*-mer hash methods. The ASV and *k*-mer hash counts were scaled to the minimum depth of each sample with the following equation (33):


$$\text{COM}\left(\omega_{i}^{\left(j\right)}\right)=\left[\text{round}\left(\omega_{1}^{\left(j\right)}\frac{m^{\left(\text{min}\right)}}{m^{\left(j\right)}}\right)\ldots ,\text{round}\left(\omega_{p}^{\left(j\right)}\frac{m^{\left(\text{min}\right)}}{m^{\left(j\right)}}\right)\;\right]\epsilon \;R^{n\times p},$$

where i=1,…,p is the ASV or *k*-mer index, j=1,…,n is the sample index, $\omega_{i}^{\left(j\right)}$ is the read count of ASV or *k*-mer i in sample j, and $m^{\left(j\right)}$ is the total ASV or *k*-mer hash count number for sample j, where $m^{\left(j\right)}=\;\sum_{i=1}^{p}\omega_{i}^{j};\;m^{\left(\text{min}\right)}=\text{min}\left\{m^{\left(1\right)},\;m^{\left(2\right)},\;\ldots ,\;m^{\left(n\right)}\right\}\;and\;\text{round}\left(\right)$ is an operator rounding the fraction to be the nearest integer.

### Data integration and the removal of confounding factors

Data integration by using the Combat function in the “sva” R package (version 3.42.0) was applied to remove batch effects or confounding effects indicated in the study’s metadata, including sample type and study of origin for the PS data sets and location and study of origin for the PQ data sets (34). The parameter “par.prior” was set as “false” to use the non-parametric adjustments, and the parameter “mean.only” was set to “true” to adjust the mean of the batch effect across batches (34).

### Classification of fresh PS and quality samples

Classifiers for PS and PQ using either the processed ASV or *k*-mer hash data sets were constructed. Three different classification methods, including RFs (35), *k*-nearest neighbors (*k*-NNs) (36), and fully connected, feed-forward neural networks (NNs) (37), were evaluated (Fig. S2 and S3). For RF, the number of decision tree (*n*_tree_) was set at 500, and the number of features randomly sampled as candidates at each split (mtry) was set to the square root of the total number of input features. RF-based models were trained by using the “randomForest” R package (38) (version 4.7-1.1). Predicted class labels were decided based on the majority vote (>50%) by 500 decision trees. The *k*-NN-based classifiers were trained by using the “caret” R package (version 6.0-94). *k* values ranging from 1 to 10 were tested in order to identify *k* values with the best classification performance (Fig. S2 and S3). The feed-forward NN was trained by using the “nnet” R package (version 7.3-19), with hyperparameters set as the same as Arbajian et al. (39) and Džal et al. (40). The “nnet” fits a feed-forward NN with a single hidden layer. The number of nodes in the hidden layer was set to 5, the decay parameter was set to 0.1, and the activation function was set to the logistic activation function.

### Model validation

For the individual data set classification experiments, we used 10-fold cross-validation to measure the accuracy of RF-, *k*-NN-, and NN-based classifiers using the “caret” R package (version 6.0-94). Unweighted total classification accuracy across both the case and control phenotypes was measured, as the classification experiments were generally well balanced (Table S1). Each experiment was conducted in five replicates. As we found RF-based models using 7-mer hash data sets had better PS or PQ classification performance than the *k*-NN- and NN-based models, the following experiments were only carried out based on the RF method.

A number of cross-study classification experiments were also conducted in order to test the generalization performance of PS and PQ classifiers. In the cross-study classification experiments for PS, we constructed pairs of training and test data sets in which one study formed the test data set and the remaining studies formed the training data set. For example, for the PS experiments, we constructed a training data set consisting of individuals or a combination of the LiaoRl21 and Zhang18 studies, while using the LiaoSm21 study as a test data set.

### Evaluation of feature importance

To evaluate and quantify the importance of individual features identified based on the ASV or the *k*-mer hash method, features were ranked by their mean decrease in accuracy (MDA) calculated by the RF-based classification model. The MDA quantifies the importance of a variable by measuring the decrease in prediction accuracy when the variable is randomly permuted in comparison with the original observations (41).

### Taxonomic analyses

To profile the bacterial communities within individual produce samples and carry out the differential abundance analysis between phenotypes, taxonomic analyses at the genus level were conducted by using the QIIME 2 (version 2022.8) pipeline for the ASV data set. The QIIME 2 plugin “q2-feature-classifier” (42) was used to assign ASV to the SILVA 138 small subunit rRNA database as the taxonomy reference (43). Chloroplast, mitochondria, and unassigned taxa were filtered by using “qiime taxa filter-seqs” command in QIIME 2 (44). For integrated taxonomic data sets, the COM method was first employed for data normalization. Data integration was carried out by using Combat to regress out study of origin and sample types for PS and study of origin and location for PQ data sets. The processed taxonomic data sets were employed for the differential abundance analysis between “contaminated” and “non-contaminated” samples and between GQ and DQ samples.

For taxonomic analysis of 16S rRNA gene sequences based on the *k*-mer hash data sets, the SILVA 138 database was first downloaded, and the taxonomic sequences were computed for 7-mer hash using the command “sourmash sketch dna” in the sourmash pipeline. The sourmash lowest common ancestor (LCA) taxonomy database was established by using a “sourmash lca index” command on the processed 7-mer hash of taxonomic sequences from the SILVA 138 database, followed by taxonomic classification of query 7-mer hash data sets against the established sourmash LCA taxonomy database carried out by using a command “sourmash lca classify” (26). However, the query *k*-mer hashes could not be correctly assigned to the sourmash LCA taxonomy database based on the SILVA database. This issue may be due to the inappropriateness of the current algorithm of LCA taxonomic classification in the sourmash pipeline for 16S rRNA gene sequences. This is an established issue (https://github.com/sourmash-bio/sourmash/issues/1421). Therefore, the taxonomic analysis here was performed only on the ASV representation of the data.

In addition to establishing RF-based models using integrated PS (IPS) and integrated PQ (IPQ) data sets in ASV and 7-mer hash formats, the integrated ASV-based taxonomic data sets and feature-selected taxonomic data sets with features only associated with positive MDA values were also used to construct RF-based models for the prediction of PS and PQ statuses. The classification performance was evaluated by comparing the classification accuracy, computing time, and computing memory usage with those from the 7-mer hash-based PS and PQ models.

### Data visualization and statistical analysis

The non-parametric Wilcoxon rank sum test was applied to test for significant differences in classification accuracy between pairs of RF-based, *k*-NN-based, and NN-based classifiers. The analysis of the composition of the microbiome with bias correction (ANCOM-BC) test and the RF feature selection method mentioned above were employed to identify the bacterial indicators that had significant abundance changes in one group over another (45). All the above statistical analyses were conducted in R (version 4.2.2).

## RESULTS

### *k*-mer hash analysis leading to more accurate classification of microbiome samples

We first tested the hypothesis that using a *k*-mer hash strategy to analyze 16S rRNA gene sequencing data that avoid the effective data loss in the ASV strategy would improve PS and PQ classification accuracy and indicator identification. Figure 1A illustrates the conceptual differences between the two strategies. For each individual PS and PQ study, we constructed ML-based classifiers by selecting one of three methods (RF, *k*-NN, and NN) and training them using either the ASV or *k*-mer hash representations of the study data. Overall, the RF-based classifiers using 7-mer hash (Fig. 1B) representations showed higher classification accuracy than *k*-NN-based and NN-based for both PS (Fig. S2) and PQ (Fig. S3). RF-based classification using the 7-mer hash representation also generally outperformed classification using the ASV representation by 8% on average, except for the LiaoSm21_PS and LiaoSm21_PQ studies (Fig. 1B). In LiaoSm21_PS, both the ASV and 7-mer hash versions of the data yielded an accuracy of 100%. To test if the trained models were overfitting, we split each of the LiaoSm21 data sets into a training set and test set at the ratios of 8:2 or 5:5. The results showed that the classification accuracy of tested models using LiaoSm21 data sets in ASV and 7-mer hash was still 100% (Fig. S4). We also found that the *k*-mer hash data sets performed better with larger *k* values within the range of 3–7. This is unsurprising because larger *k* values lead to more features extracted from the sequencing data, which in turn provides more opportunities to distinguish DNA sequence signatures between cases and controls.

### Integrated microbiome data analysis leading to more generalizable classification

**Fig 2:**
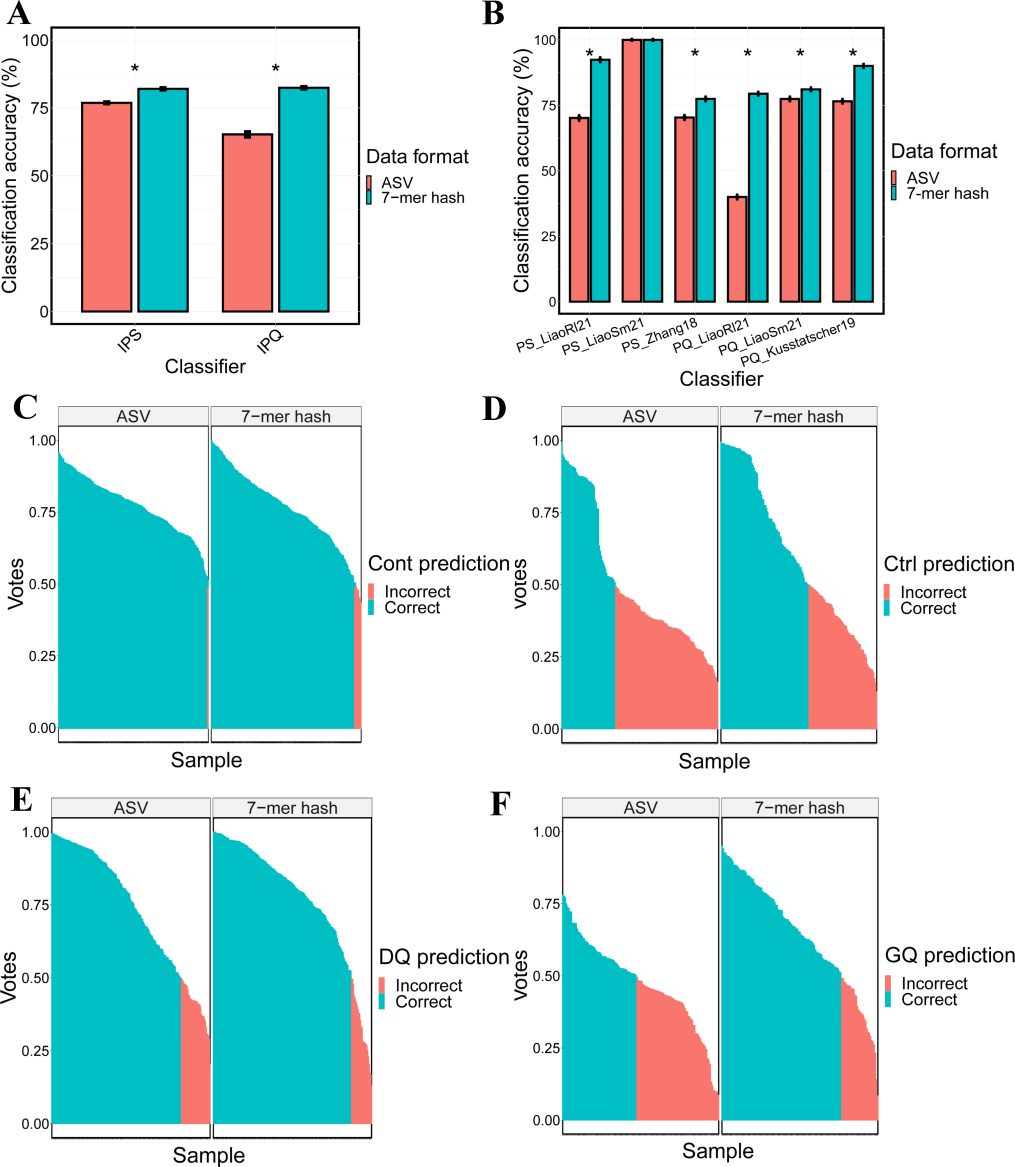
Evaluation of RF-based IPS and IPQ classifiers. (**A**) Barplot of classification performance of RF-based models on the IPS and IPQ data sets, represented using either ASV or 7-mer hashes. The Wilcoxon rank sum test was used for the pairwise comparison of the accuracies of the IPS and IPQ classifiers. (**B**) Barplot of classification performance of classifiers established by using individual data sets from IPS and IPQ data sets in ASV and 7-mer hash. (**C**) Barplot of the prediction of PS samples with the true label of contamination. The prediction was made by votes of 500 decision trees in RF-based classifiers established by using ASV and 7-mer hash. The cutoff voting rate (50% votes) indicates whether a labeled sample is predicted correctly or not. Ctrl and Cont represent non-contaminated samples and contaminated samples. The * stands for *P* < 0.05. (**D**) Same as **C**, but barplot of the prediction of PS samples with the true label of control. (**E**) Same as **C**, but barplot of the prediction of PQ samples with the true label of DQ. (**F**) Same as **C**, but barplot of the prediction of PQ samples with the true label of GQ.

To increase the effective sample sizes of published microbiome studies and therefore identify taxa that are robustly associated with PS and PQ, a single IPS data set and a single IPQ data set were established by applying batch correction and merging studies within each category. We computed ASV and 7-mer hash representations of the IPS and IPQ data sets and used them to construct RF-based models whose classification performance was compared against our previous models constructed on individual studies. Consistent with the analyses conducted on the individual studies, the integrated 7-mer hash representation achieves higher classification accuracy compared to ASV representation by 5% and 17% for the IPS and IPQ data sets, respectively (Fig. 2A), supporting the notion that the 7-mer hash data set retains more actionable information in sequencing reads. Figure 2B illustrates the performance differences separated by component study, and we see that the classification performance of the 7-mer hash representation is systematically higher in samples from all studies, not just a selected study. We also found that for the IPS data set, when we replaced the binary labels with the pathogen-specific labels (*E. coli* O157:H7, *L. monocytogenes*, and *Salmonella* Infantis), performance similarly was higher for 7-mer representations (85%) compared to ASV (78%) (Fig. S5). The accuracy of the 7-mer hash IPS classifier was 82% for the PS classification, and when the IPS classifier was used to predict specific pathogens, it rose to 85% (Fig. S5). Our IPS classifier, therefore, performs as well as the commonly used culture-dependent approach using selective agars that does not rely on sequencing (46). Given that the size and number of PS data sets will increase over time, we expect sequencing base classifiers to outperform the culture-dependent approach in the future. For the IPQ classifiers that achieved 82% accuracy, there are no benchmarks to compare their performance against as this was the first time that predictive models were used to evaluate the quality of fresh produce using microbiota and identify microbial indicators associated with GQ and DQ of fresh produce.

To gain insight into which samples were better classified under the integrated 7-mer hash data set, we then visualized the label predictions of individual samples generated by the RF-based classifiers. Figure 2C through F compares the voting rates based on ASV and 7-mer hash for predicted versus true labels of each sample in IPS and IPQ. For the IPS samples, while the number of correctly predicted contaminated samples is similar (254 and 264 for 7-mer hash and ASV, respectively) (Fig. 2C), there is a marked increase in accuracy for 7-mer hash predictions of the non-contaminated samples (85 and 52 for 7-mer hash and ASV, respectively) (Fig. 2D). Similarly, in the analysis of the IPQ samples, the 7-mer hash representations led to similar prediction of the DQ samples (174 and 163 for 7-mer hash and ASV, respectively) (Fig. 2E), while the number of correctly predicted GQ samples was higher for 7-mer hash predictions (100 versus 62 for 7-mer hash and ASV, respectively) (Fig. 2F). Our results support our hypothesis that the 7-mer hash representations lead to better classification performance of microbiome samples, consistent with our results on individual samples.

**Fig 3:**
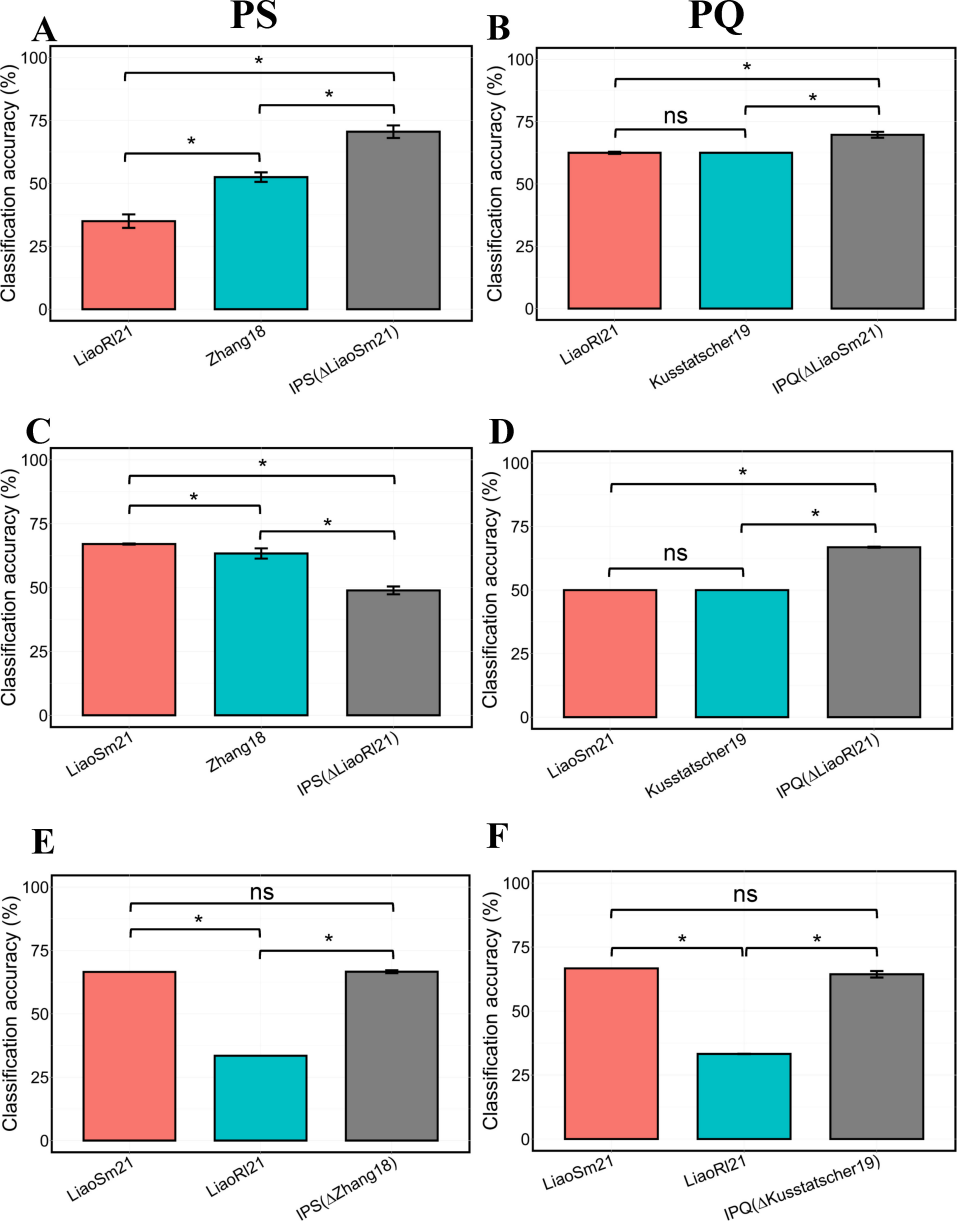
Comparison of the classification performance of classifiers trained on individual PS and PQ data sets and IPS and IPQ data sets. (**A**) Classifiers were trained on individual LiaoRl21 or Zhang18 data sets, and a classifier was trained on an integrated data set excluding LiaoSm21, denoting IPS (∆LiaoSm21). The LiaoSm21 was used as a testing set. (**B**) Classifiers were trained on individual LiaoRl21 and Kusstatscher data sets and an integrated data set excluding LiaoSm21, denoting as IPQ (∆LiaoSm21). The LiaoSm21 was used as a testing set. (**C**) Same as **A**, but individual and IPS classifiers were tested on LiaoRl21. (**D**) Same as **B**, but individual and IPQ classifiers were tested on LiaoRl21. (**E**) Same as **A**, but individual and IPS classifiers were tested on Zhang18. (**F**) Same as **B**, but individual and IPQ classifiers were tested on Kusstatscher19. Wilcoxon rank sum tests were applied for testing the significance of the difference in testing accuracy (%) of individual and IPS and IPQ classifiers. PS and PQ mean produce safety and produce quality, respectively. The * represents *P* < 0.05; the ** stands for *P* < 0.01; and the ns represents no significance.

We next wondered to what extent the integration of microbiome data from multiple studies explicitly led to the construction of more generalizable classifiers, compared to classifiers trained on individual studies. We, therefore, performed six experiments (three for each of IPS and IPQ), in which we repeatedly removed one study as a test (held-out) study, and compared the model performance of RF classifiers when trained on the remaining two studies separately versus combined (Fig. 3). In principle, classifiers trained on the combined 7-mer hash data sets would be encouraged to learn features that are more broadly associated with either produce pathogen contamination or PQ decline, because both the case and control samples in the combined data set will be more heterogeneous compared to the samples in the individual studies. Figure 3 shows that across the six experiments, the integrated data sets performed significantly better than the individual data sets alone in three of them (Fig. 3A, B and D), achieving on average 17% higher accuracy than individual data sets. In comparison, for only one experiment, combining data led to significantly (*P* = 0.012) worse performance based on non-parameter Wilcoxon rank sum test (Fig. 3C). For Fig. 3C, RF models were trained using data sets of LiaoSm21, Zhang18, and IPS without the LiaoRl21, respectively. The training classification accuracy of the RF-based models was 100%, 75%, and 82% (Fig. S6A), which decreased to 67%, 63%, and 49% for the testing classification accuracy of the models shown in Fig. 3C. The MDA-positive features that contribute to the PS classification of the models using individual data sets and that of the model using integrated data sets were compared. LiaoSm21 has a much lower number of MDA-positive features (343 versus 3,255). Among the MDA-positive features, there are 198 shared features, and LiaoSm21 has 145 unique ones (Fig. S6B). For Zhang18, the number of MDA-positive features was also lower than that of the integrated data set (2,713 versus 3,255). Among these features, 1,204 features are shared, and Zhang18 has 1,509 unique features (Fig. S6B). The unique MDA-positive features in each single data set could be a reason for causing the testing accuracy of the single data set-trained model higher than that of the integrated data set-trained model. The unique features with higher MDA values play a more important role in the PS or PQ prediction (Table S2). These results suggest that it can be sensible to combine data from multiple studies containing case and control samples together, which tend to lead to better generalizable performance of the classifiers for both PS and PQ.

### Integrated taxonomic analysis identifying generalizable taxa associated with PS and quality

The higher classification performance of the integrated data sets suggests that there are taxa that are broadly associated with PS and PQ identified by the classifiers. We analyzed taxonomy based on the ASV representation of the IPS and IPQ data sets. Subsequently, we trained RF models on the ASV-based taxonomy data sets for predicting PS and PQ status and then compared their classification performance with that of 7-mer hash-based models.

**Fig 4:**
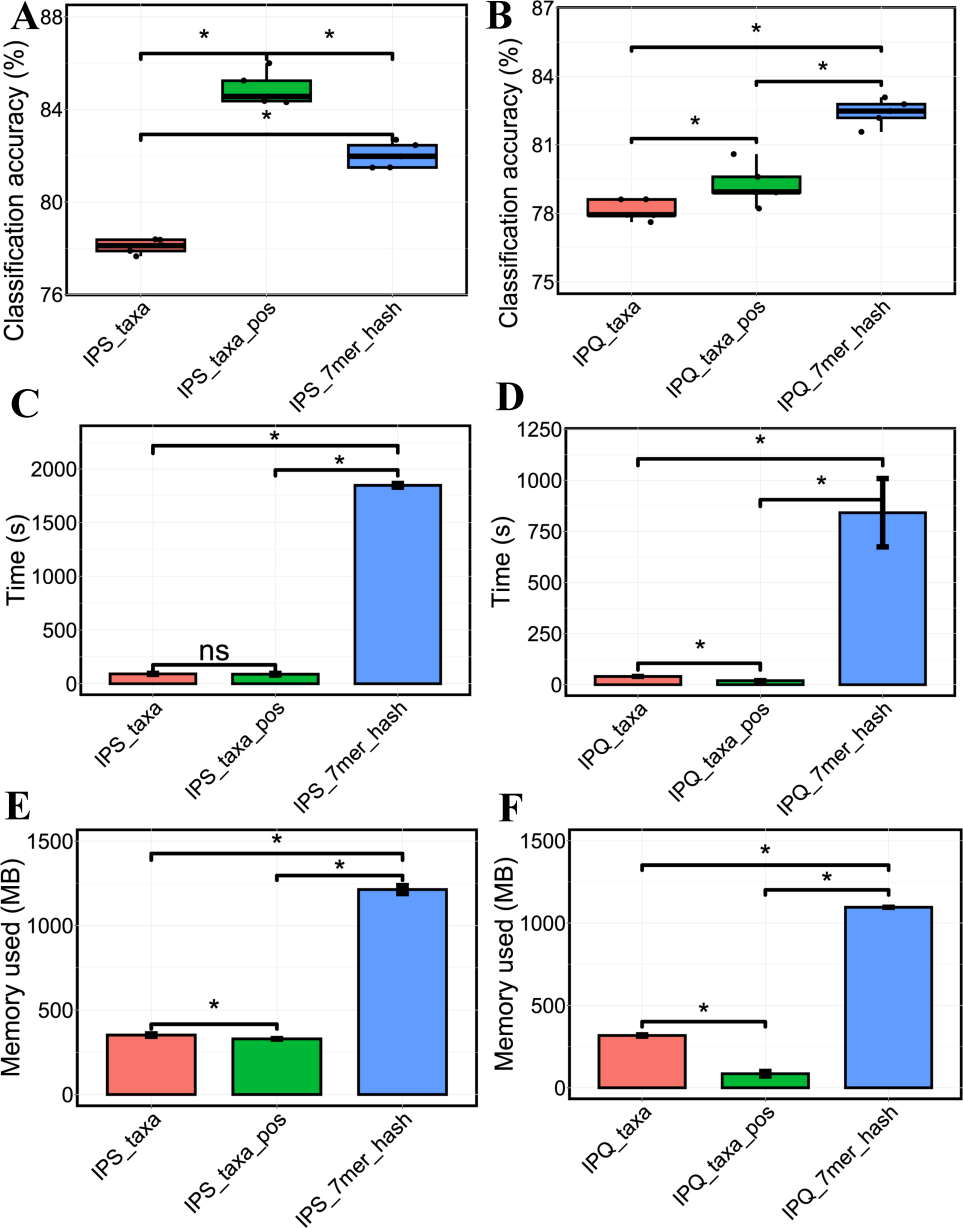
Comparison of PS and PQ classification performance between RF-based models constructed using ASV-based taxonomic data sets and 7-mer hash data sets. (**A** and **B**) PS and PQ classification accuracy of models using ASV-based taxonomic data sets, feature-selected taxonomic data sets with positive MDA, and 7-mer hash data sets, respectively. (**C** and **D**) Computing time (s) of PS and PQ classification by the models using the three types of data sets mentioned above. (**E** and **F**) Computing memory usage (MB) of PS and PQ classification by the models. “*” strands for *P* < 0.05, indicating significant differences present between groups of samples. MB, megabyte; s, second.

The PS and PQ classification performance between RF-based models constructed using ASV-based taxonomic data sets and 7-mer hash data sets was evaluated based on classification accuracy, computing time, and computing memory usage. For classification accuracy, the models trained on the feature-selected taxonomic data sets with positive MDA values had significantly higher PS classification accuracy than those based on 7-mer hash (Fig. 4A), but the selected taxonomy-based models had lower PQ classification accuracy compared to the 7-mer hash-based models (Fig. 4B). The models trained on the feature-selected taxonomic data sets had significantly higher classification accuracy for both PS and PQ than the models trained on the whole taxonomic data sets. For the computing time, the ASV-based taxonomy strategy spent significantly less time than the 7-mer hash strategy for both PS classification (Fig. 4C) and PQ classification (Fig. 4D). Within the taxonomy strategies, the models based on the feature-selected taxonomic data sets saved more time for PQ classification compared to the models based on the full taxonomic data sets. Similarly, for computing memory usage, the taxonomy strategy used remarkably less memory than the 7-mer hash strategy for both PS classification (Fig. 4E) and PQ classification (Fig. 4F). The models based on the feature-selected taxonomic data sets used less memory than that based on the full taxonomic data sets for both PS classification and PQ classification (Fig. 4E and F).

For the IPS taxonomic analysis, 1,357 genera were identified in total. The most dominant genus in the bacterial communities across fresh produce samples was *Pseudomonas* (with relative abundance ranging from 0.07% to 57.95%), followed by *Flavobacterium* (1.31% to 41.31%). Among the genera, 226 unclassified genera were identified. The total relative abundance of the unclassified genus group across samples ranged from 4.54% to 43.61%. The most abundant unclassified genus under Comamonadaceae (0.0012%–24.06%) in this group ranked as the fifth largest taxa.

**Fig 5:**
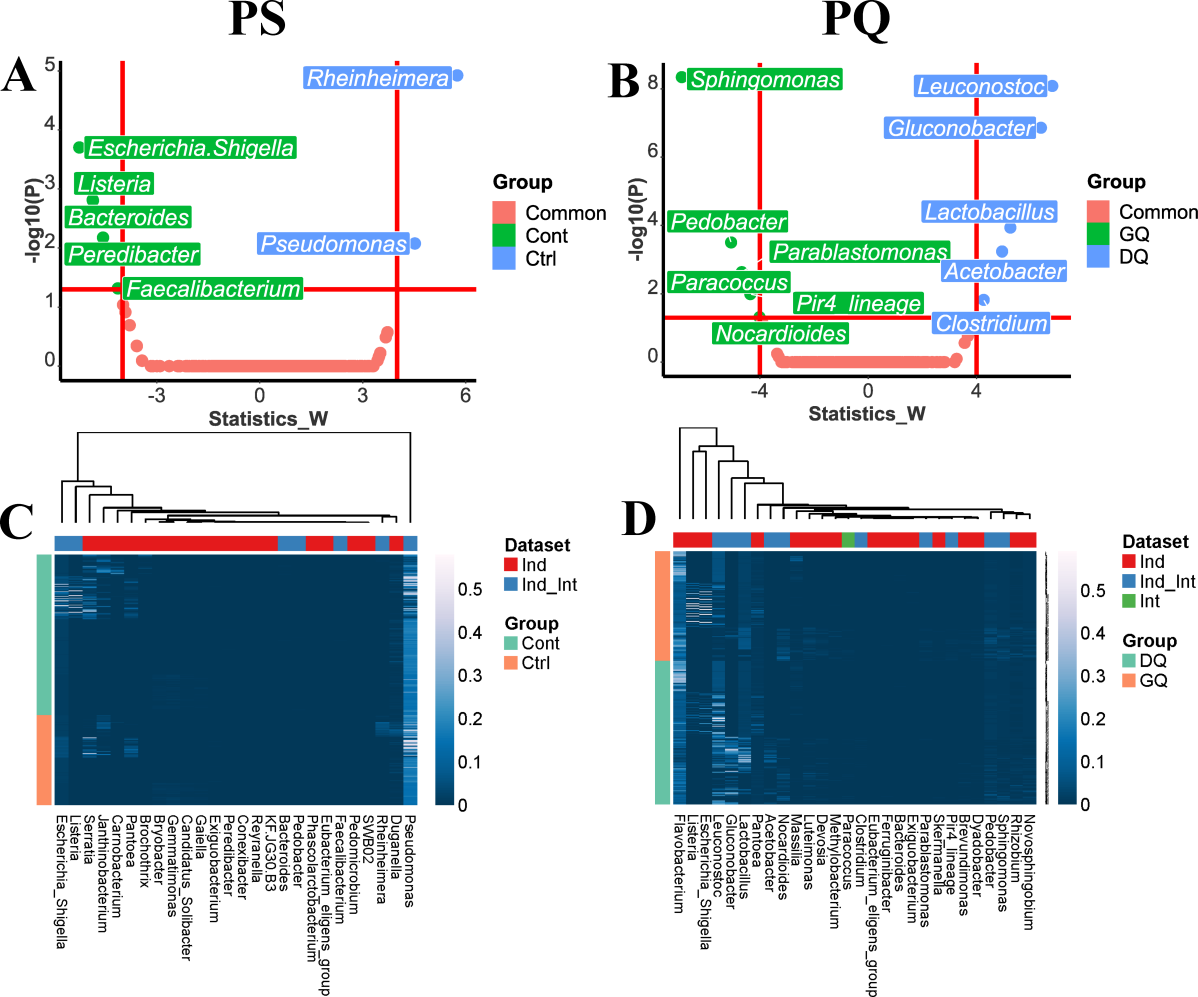
Identification of bacterial indicators related to PS or PQ. (**A** and **B**) Volcano plots based on the *W* statistic values and −log10(*P*) values obtained from the ANCOM-BC test presenting the differential abundances of genera among two contamination groups for PS and PQ, respectively. (**C** and **D**) Heatmaps of the relative abundances of bacterial indicators identified from individual and IPS data sets and individual and IPQ data sets, respectively. Cont means contaminated samples. Ctrl represents non-contaminated samples. DQ represents decreasing-quality samples. GQ represents good-quality samples.

The ANCOM-BC test was applied to identify bacteria at the genus level that have significantly different relative abundance between contamination groups (contaminated samples versus non-contaminated samples) or pathogen groups (*E. coli* O157:H7, *L. monocytogenes*, and *Salmonella* Infantis). Five genera were identified as indicators for the contaminated group, including *Escherichia-Shigella*, *Listeria*, *Bacteroides*, *Peredibacter*, and *Faecalibacterium*, and two indicators (*Rheinheimera* and *Pseudomonas*) were identified for the non-contaminated group (Fig. 5A). Their significance values were listed in Table S3. We noticed that the contaminating pathogens, *E. coli* O157:H7 and *L. monocytogenes*, were identified as *Escherichia-Shigella* and *Listeria*. Our previous study (11) reported that no *Escherichia* could be identified in contaminated spring mix samples when the *E. coli* O157:H7 inoculation level was at 5.5 Log CFU/mL by using 16S rRNA gene sequencing. The authors assumed that the concentration of *E. coli* O157:H7 was below the limit of detection of 16S rRNA gene sequencing method. However, the present study showed that *Escherichia-Shigella* was identified for *E. coli* O157:H7 with relative abundance from 0.019% to 0.18%. One explanation for the difference is that the present study conducted the taxonomic analysis based on the SILVA 16S rRNA sequence database, while Liao and Wang (11) applied the Greengenes database, which contains less annotated taxonomic references than the SILVA database (11).

On the other hand, the *Salmonella* Infantis was not identified using the Zhang18 study (12). One explanation is that 10^4^ copies/mL of *Salmonella* Infantis located in the root of lettuce was below the limit of detection of 16S rRNA gene sequencing. In this study, *Peredibacter* was identified as an indicator for *Salmonella* Infantis-contaminated samples although *Salmonella* Infantis could not be detected. Our result indicates that *Bacteroides* could be also applied as pathogen contamination indicator for produce. Davidov and Jurkevitch (47) reported that *Peredibacter* is a member of *Bdellovibrio*-and-like organisms that are highly motile microbes preying on other Gram-negative bacteria. Lu and Cai (48) reported that *Peredibacter* sp. strain BD2GS significantly retarded the growth of *Salmonella* Typhimurium within 3–12 hours through lysing prey cells (48). Based on these, the elevated relative abundance of *Peredibacter* might be triggered by contamination of *Salmonella* Infantis. *Bacteroides* is an obligate anaerobic bacterium making up a remarkable portion of fecal bacterial communities, which has been suggested to be used as fecal indicator organisms for water samples (49). *Bacteroides* and *Faecalibacterium* have been reported as commensal bacteria of the human gastrointestinal microbiota (50, 51) and classified as fecal indicator bacteria (52). Savichtcheva et al. (53) reported that 16S rRNA gene marker of *Bacteroides* had a better prediction for the presence of bacterial enteric pathogens than total and fecal coliforms (53).

For the taxonomic analysis at the genus level of integrated microbiome data sets related to PQ, 760 genera were identified. The most relatively abundant genus was *Pseudomonas* (0.10%–58.84%), followed by *Flavobacterium* (0.73%–38.53%). Among the genera, 97 unclassified genera were identified. The total relative abundance of the unclassified genus group across samples ranged from 5.49% to 55.52%. The most relatively abundant unclassified genus under Yersiniaceae (0.015%–45.9%) in this group ranked as the 10th largest taxa. Through the ANCOM-BC test, five genera, including *Leuconostoc*, *Gluconobacter*, *Lactobacillus*, *Acetobacter*, and *Clostridium*, were identified as indicators in DQ samples (Fig. 5B). Their significance values were listed in Table S4. Del Árbol et al. (54) mentioned that *Gluconobacter* and *Acetobacter* are acetic acid bacteria, which can generate acetic acid to spoil fruits causing bacterial rot and browning. *Leuconostoc* and *Lactobacillus* are commonly known as psychrotrophic spoilage lactic acid bacteria that spoil meat products and fresh fruits and vegetables during 4°C storage (55, 56). *Clostridium* spp. have also been reported to be associated with meat and cheese spoilage (57, 58). In the Kusstatscher et al. (9) study, except for *Clostridium*, the other four genera identified as indicators in DQ samples in the present study were recognized as core microbiota in decaying samples. Six genera, including *Sphingomonas*, *Pedobacter*, *Parablastomonas*, *Paracoccus*, *Pir4_lineage*, and *Nocardioides*, were identified as indicators in GQ samples. These six indicators for GQ samples were reported to be related to agriculture rhizosphere soil (59–64). Three genera, *Sphingomonas*, *Pedobacter*, and *Nocardioides*, were also identified as core microbiota in healthy samples in the Kusstatscher et al. study (9).

Most of the indicators identified in the integrated data sets were covered by the indicators identified from individual data sets (Fig. S7 and S8). For the PS-related data sets, seven indicators for the contaminated group identified from the individual data sets (Fig. S7) were also identified in the integrated data set. However, seven indicators identified for the contaminated group in the individual data sets were not identified in the integrated data set, including *Eubacterium*, *Janthinobacterium*, *Serratia*, *Carnobacterium*, *Phascolarctobacterium*, *Brochothrix*, and *Exiguobacterium*. Twelve indicators identified for the non-contaminated group in the individual data sets were not identified in the integrated data set including *KF.JG30.B3*, *Gemmatimonas*, *Candidatus*_*Solibacter*, *SWB02*, *Conexibacter*, *Pedomicrobium*, *Bryobacter*, *Reyranella*, *Gaiella*, *Duganella*, *Pedobacter*, and *Pantoea*. (Fig. 5C).

For PQ data sets, 10 indicators identified in the integrated data set were covered by the indicators identified in the individual data sets (Fig. S8). Interestingly, *Paracoccus* was identified as a new indicator for the GQ group only in the integrated data set. To the best of our knowledge, *Paracoccus* has not been reported to be associated with PQ. However, this genus contains a number of species that can produce astaxanthin (65), which has been reported to exhibit antagonism against food spoilage bacteria (66). Fourteen bacteria identified as indicators for the GQ group in the individual data sets were not identified in the integrated data set, including *Flavobacterium*, *Ferruginibacter*, *Devosia*, *Skermanella*, *Luteimonas*, *Methylobacterium*, *Novosphingobium*, *Dyadobacter*, *Bacteroides*, *Exiguobacterium*, *Pantoea*, *Eubacterium*, *Escherichia-Shigella*, and *Listeria*, and four indicators recognized for the DQ group in the individual data sets were not identified in the integrated data set, including *Brevundimonas*, *Rhizobium*, *Pedobacter*, and *Dyadobacter* (Fig. 5D).

In addition to identifying critical features associated with PS and PQ using the ANCOM-BC test, we ranked the features based on the MDA measures from RF-based PS and PQ classifiers established using individual and integrated ASV or 7-mer hash data sets and classified them into three groups, including negative, zero, and positive contributions to PS and PQ classification (Fig. S9; Table. S5). RF-based classifiers using the 7-mer hash representation utilized on average 62% of the 8,192 hash features provided to RF for the integrated data sets, compared to an average of 9% of the ASV features provided to RF for the integrated data sets (Table S5). Our interpretation is that the 7-mer hash representation may lead to better classification performance in part because so many features are leveraged in the classification; this may make classification more robust by making individual feature weightless toward the label prediction.

**Fig 6:**
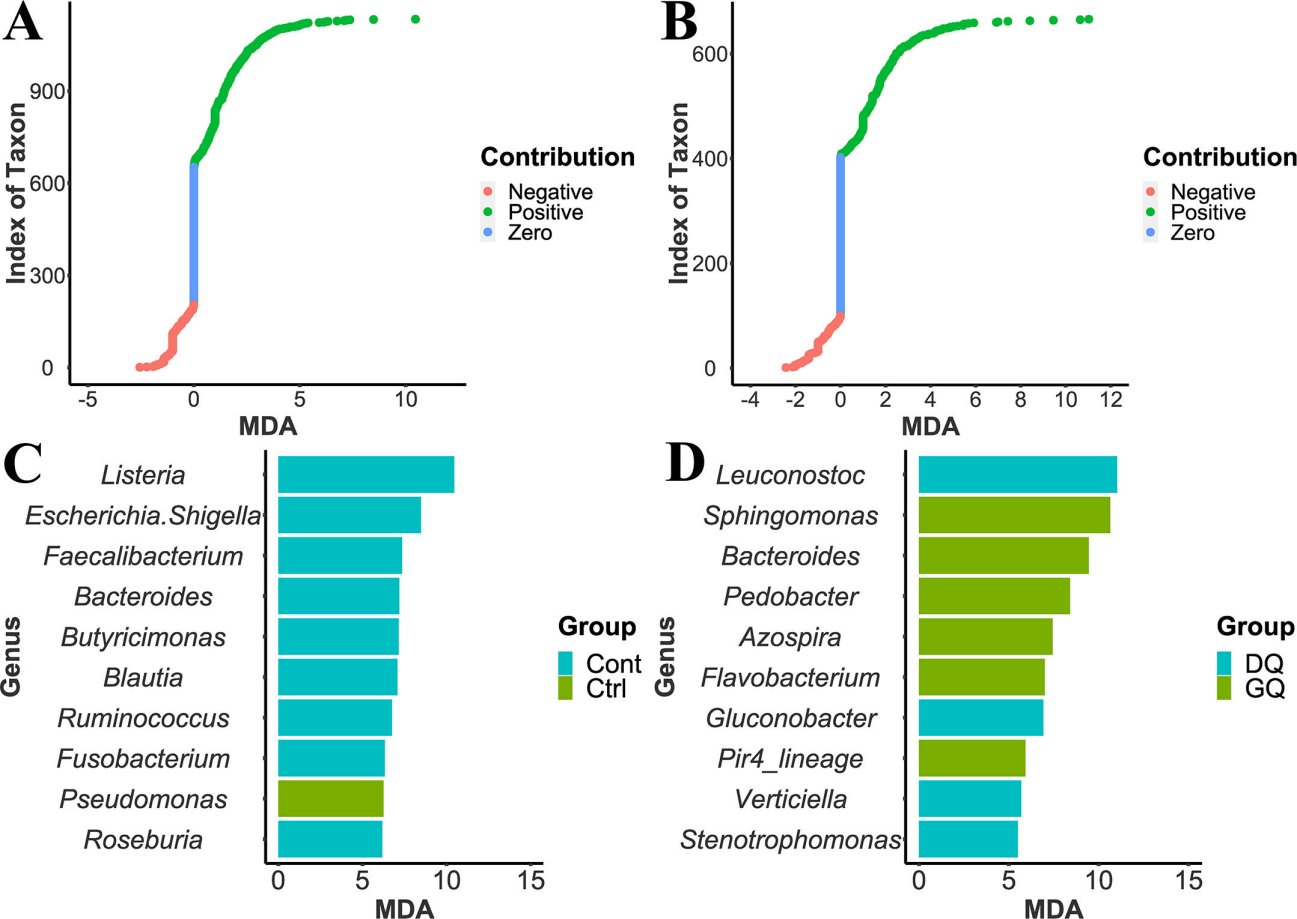
Importance of features evaluated by MDA provided by RF-based classifiers established using ASV-based taxonomy strategy. (**A**) Contribution of taxonomic features to PS classification. (**B**) Contribution of taxonomic features to PQ classification. (**C**) The top 10 most important genera with a positive contribution to IPS classification. (**D**) The top 10 most important genera with a positive contribution to IPQ classification. Ctrl represents the non-contaminated group, and Cont represents the contaminated group. GQ represents good quality, and DQ represents decreasing quality.

In addition, we evaluated the importance of identified genera contributing to PS or PQ classification through MDA measures from RF-based models using ASV-based taxonomy. In summary, the identified IPS genera contained 480 genera with a positive contribution, 205 genera with a negative contribution, and 447 genera with zero contribution to PS classification (Fig. 6A). The identified IPQ genera covered 263 genera with a positive contribution, 100 genera with a negative contribution, and 302 genera with zero contribution to PQ classification (Fig. 6B). These genera with a positive contribution were regarded as the potential indicators related to PS and PQ classification. Among the IPS positive-contributing genera, 194 were considered contamination indicators, and 287 were considered non-contamination indicators. The top 10 most important genera for PS classification were *Listeria*, *Escherichia-Shigella*, *Faecalibacterium*, *Bacteroides*, *Butyricimonas*, *Blautia*, *Ruminococcus*, *Fusobacterium*, *Pseudomonas*, and *Roseburia* (Fig. 6C). Except for *Pseudomonas*, the nine genera were identified as contamination indicators. In comparison, the PS-related indicators identified by the RF feature selection method covered all the indicators identified by the ANCOM-BC. The top 10 most important genera included four contamination indicators, *Escherichia-Shigella*, *Listeria*, *Bacteroides*, and *Faecalibacterium*, and one non-contamination indicator, *Pseudomonas*, were identified by the ANCOM-BC method. In addition, five indicators, *Butyricimonas*, *Blautia*, *Ruminococcus*, *Fusobacterium*, and *Roseburia*, were not identified by ANCOM-BC. Our previous study (11) has reported that *Fusobacterium* was identified as an indicator for *E. coli* O157:H7 contamination of spring mix salad. The other four indicators have not been reported as related to food contamination. However, previous research has reported that all of them are anaerobic bacteria that inhabit the gastrointestinal tract of humans (67–70). Our results indicate that the presence of foodborne pathogens can induce the growth of these gut microbes on fresh produce.

Among the IPQ positive-contributing genera, 109 were considered DQ indicators, and 154 were considered GQ indicators. The top 10 most important contributors to PQ classification were *Leuconostoc*, *Sphingomonas*, *Bacteroides*, *Pedobacter*, *Azospira*, *Flavobacterium*, *Gluconobacter*, *Pir4_lineage*, *Verticiella*, and *Stenotrophomonas* (Fig. 6D). Among them, *Leuconostoc*, *Gluconobacter*, *Verticiella*, and *Stenotrophomonas* were recognized as DQ indicators, and the other six were GQ indicators. In comparison, all the PQ-related indicators identified by the ANCOM-BC method were included in those from the RF feature selection. The top 10 most important genera covered two DQ indicators, *Leuconostoc* and *Gluconobacter*, and three GQ indicators, *Sphingomonas*, *Pedobacter*, and *Pir4_lineage*, were also identified by the ANCOM-BC method. In addition, two DQ indicators, *Verticiella* and *Stenotrophomonas*, and three GQ indicators, *Bacteroides*, *Azospira*, and *Flavobacterium*, from the 10 top genera were not identified by the ANCOM-BC. *Verticiella*, *Stenotrophomonas*, and *Azospira* have been reported to be present in fruit, plants, or soil (71–73), but their relation to PQ was still unclear. Our results filled the knowledge gap to illustrate the importance of *Verticiella* and *Stenotrophomonas* during quality decline and that of *Azospira* in GQ indication. Our above result indicates that *Bacteroides* as fecal indicator organisms for water samples could be also applied as a pathogen contamination indicator for produce. Although our result indicates that *Bacteroides* contribute to GQ, we may consider *Bacteroides* more to be a contamination indicator. Previous research reported *Flavobacterium* as the dominant genera in bagged spring mix salad and lettuce (11, 74). Interestingly, *Flavobacterium* has been reported to be associated with the spoilage of meat, milk, and seafood (75), while our result indicates that *Flavobacterium* contributes to the GQ of produce.

## DISCUSSION

The IPS and IPQ classifiers using 7-mer hash data sets had significantly higher accuracy than the models using ASV data sets. Two reasons are proposed: first, to obtain the ASV representations, the DADA2 plugin in QIIME 2 was used to process the primer-removed sequences, including quality filtering, denoising, chimera removing, dereplicating, and/or pair-end sequence joining. Although the parameters “--p-trim-left” and “--p-trunc-len” were set as 0 to trim or truncate no base due to the median quality score of bases at each position across all reads greater than 30 in this study, 1.66%–40.94% of sequences from the integrated data set related to PS were still discarded (Fig. S10A), and 0.50%–46.98% of overall sequences from integrated data set related to PQ were discarded (Fig. S10B). The discarded reads may contain abundant sequence variation information, which contributed to the downstream PS and PQ classification. For the *k*-mer hash representations, the raw sequences were directly used to generate the *k*-mer hash data sets by using the sourmash pipeline as all the median phred quality scores of nucleotides in reads were greater than 30 (31). Werner et al. (25) reported that 43% of the total 16S rRNA gene sequences were discarded after the denoising process (25). Second, the 7-mer hash computed from the original reads could improve the detection sensitivity of bases than ASV in a longer size (up to 421 bp). The raw sequences were subsequenced into seven-base subsequences (7-mer), then transformed into hash codes, and finally randomly picked for 8,192 of 7-mer hash signatures for each sample (26). The 7-mer hash data sets for establishing classification models can significantly shorten the computing time and improve the sensitivity of detection of different nucleotides among sequences compared with ASV. To confirm that the *k*-mer method enhances accuracy, the un-denoised ASV data sets were also obtained during the denoising step by tuning the parameters “--p-chimera-method” from “consensus” to “none” and “--p-max-ee” from the default setting 2 to the longest read length in the “qiime dada2 denoise-single” command, indicating no chimeras and erroneous bases removal. Subsequently, the un-denoised ASV-based IPS and IPQ data sets were applied to construct RF-based models. Figure S11A and B shows the 7-mer hash-based models present significantly higher (*P* = 0.012 for the IPS models and *P* = 0.016 for the IPQ models) classification accuracy than the un-denoised ASV-based models, suggesting that the 7-mer hash data sets containing shorter features are more effective for PS or PQ classification due to the increased sensitivity. In addition, the models trained on un-denoised ASV data sets had a significantly higher (*P* = 0.011 for the IPS models and *P* = 0.012 for the IPQ models) classification accuracy than that using denoised ASV data sets, suggesting that the discarded reads after denoising contained effective features contributing to the PS or PQ classification. Moreover, the RF-based models trained on integrated 7-mer hash data sets spent significantly less computing time (Fig. S11C and D) and used remarkably smaller computing memory (Fig. S11E and F) for the PS or PQ classification than that trained on the denoised ASV data sets or un-denoised ASV data sets.

The 3-mer to 7-mer hash data sets of individual studies related to PS or PQ were computed by using the sourmash pipeline, which was used subsequently to establish PS and PQ classifiers. Generally, the RF-based models using 6-mer and 7-mer hash data sets had similar accuracy but significantly higher accuracy than that of models using 3-mer to 5-mer hash data sets, except for PQ models using 3-mer of LiaoRl21. Sourmash pipeline generates *k*-mer minhash signatures of DNA sequences, which randomly samples *k*-mer content to produce small subsets as known as “sketches” (26). The Jaccard similarity of two sketches of sequence data sets remains approximately equal to their true Jaccard similarity (76). The factor “scaled” was set as 1 in “sourmash sketch dna” command to generate the hash signature without downsampling the number of sketches. Using “sourmash sketch dna” command with the *k*-mer size parameter set as 3–7 generated 32, 136, 512, 2,080, and 8,192 *k*-mer hashes of each DNA sample, respectively. Based on this, 7-mer hashes with a larger size than 3-mer to 6-mer contain more sequence variances, which potentially contribute to the higher classification accuracy of models (77). In addition, a larger *k* size of *k*-mer improves the specificity of different bases among sequences (78). However, when *k* was set greater than 7, for example, when *k* = 8, there were 32,896 of 8-mer hashes computed. The computing time and memory usage remarkably increased while the accuracy of models had no obvious improvement (data not shown).

Although our results showed 7-mer hash-based models had better classification performance for PS and PQ than ASV-based models, a current drawback of this method is that taxonomy analysis based on 16S rRNA gene sequencing data in 7-mer format is still unavailable, which may be caused by the inappropriate LCA algorithm for the taxonomy analysis. In addition, although reducing the effective read loss without using the denoising step in 7-mer hash preprocessing improved the PS and PQ classification performance of RF-based models, it may decrease the accuracy of taxonomy identification due to noising reads. The trade-off between saving effective reads and ridding noising reads may need to be explored. Due to these considerations, we also constructed the RF-based models for PS and PQ classification using ASV-based taxonomic data. To compare with 7-mer hash strategy, the models constructed using feature-selected ASV-based taxonomy presented significantly better classification performance for PS and a bit lower classification accuracy for PQ. However, its computing time and memory usage were remarkably smaller, indicating that the ASV-based taxonomy strategy can be more efficient and applicable for PS and PQ classification and identification of important associated indicators.

On the basis of the taxonomic analysis, we identified Pseudomonas and Flavobacterium as dominant genera in PS- and PQ-related samples, which have been previously reported to be predominant in bagged spring mix salad and lettuce (11, 74). However, unclassified genera with high relative abundance were not often mentioned in fresh produce microbiota studies. The integration of data sets might bring out more unclassified genera than individual data sets, due to the limited number of taxonomic references available for taxonomic analysis. In the previous PS and PQ studies, a few bacterial indicators for produce contamination (11) or decaying produce (9) have been identified. However, these indicators were not consistent across the individual data sets due to the limited data size. In this study, we applied the data integration method to homogenize three PS data sets and three PQ data sets, respectively. The ANCOM-BC test was then employed to identify indicators for pathogen contamination and quality reduction of fresh produce based on individual data sets and integrated data sets. We identified 26 genus indicators and 28 genus indicators for PS and PQ produce, respectively, from individual data sets. Among them, seven indicators related to PS and 10 indicators related to PQ were validated by the ANCOM-BC test using the integrated data sets. These validated indicators can provide a more generalizable and consistent indication or prediction of PS or PQ statuses (79). Interestingly, we also identified a new indicator, *Paracoccus*, for GQ produce only from the IPQ data set. The result indicates that more new indicators could be potentially identified with the size increase of integrated data sets. The non-validated indicators from individual data sets may be due to these studies having different types of fresh produce, distinct inoculated pathogens, and/or various storage conditions, which make the composition and diversity of bacterial communities largely different.

In addition to the ANCOM-BC test, the RF feature selection method was also used to identify indicators for fresh produce contamination and quality reduction. Compared to the indicators identified by the ANCOM-BC test, we found that RF feature selection method can identify much more indicators for PS (480 genera) and PQ (263 genera) with a positive contribution to the PS and PQ classification. These features covered all the indicators identified by the ANCOM-BC test, indicating that the RF-based models are more sensitive and powerful to catch the variation of features between classification groups. RF feature selection method and ANCOM-BC test can be used together to determine the reliable indicators for indicating PS and PQ. Sheh et al. (80) reported that RF-based models were the most accurate models and correctly classified strictures for chronic gastrointestinal diseases using nine ASVs. Based on the RF-based model and ANCOM results, *Clostridium perfringens* was identified as a potential causative agent associated with the development of strictures.

### Conclusion

In summary, we established and compared RF-based PS and PQ classifiers by using publicly available microbiome data sets in ASV and 7-mer hash for predicting the contamination conditions or quality statuses. This study illustrates that the 7-mer hash-based approaches are useful for building more accurate classifiers than ASV but not necessarily for the taxonomic analysis yet. Due to this current limitation, we also explored an ASV-based taxonomy strategy for PS classification and PQ classification, which performed significantly better than the 7-mer hash strategy for PS classification and were remarkably more computing-efficient. Data integration of multiple data sets leads to greater classification performance of the integrated RF-based models than that using individual data sets, with significantly higher accuracy and more features with positive contribution to PS or PQ classification identified. In addition, we found that more consistent and generalizable microbes were identified as indicators for safety and quality groups of fresh produce through integrated taxonomic analysis, illustrating the benefits of integrating data sets.

## Data Availability

Raw reads from this study have been deposited to the National Center for Biotechnology Information under the project accession number PRJNA792031. The sample data set is available in the GitHub repository (https://github.com/LZC0034/CeDAR-Project). The codes for data processing are available in the GitHub repository (https://github.com/LZC0034/CeDAR-Project).

## SUPPLEMENTAL MATERIAL

Supplemental figures spectrum.03448-23-s0001.docx Fig. S1 to S11.

Supplemental tables spectrum.03448-23-s0002.docx Tables S1 to S5.

ASM does not own the copyrights to Supplemental Material that may be linked to, or accessed through, an article. The authors have granted ASM a non-exclusive, world-wide license to publish the Supplemental Material files. Please contact the corresponding author directly for reuse.
