# Supplementary material for: Microbiome-based classification models for fresh produce safety and quality evaluation
Source: Microbiol Spectr. 2024 Mar 6;12(4):e03448-23. doi: 10.1128/spectrum.03448-23 (PMC10986475; doi:10.1128/spectrum.03448-23)
Supplement: Supplemental figures — Fig. S1 to S11. [file spectrum.03448-23-s0001.docx]

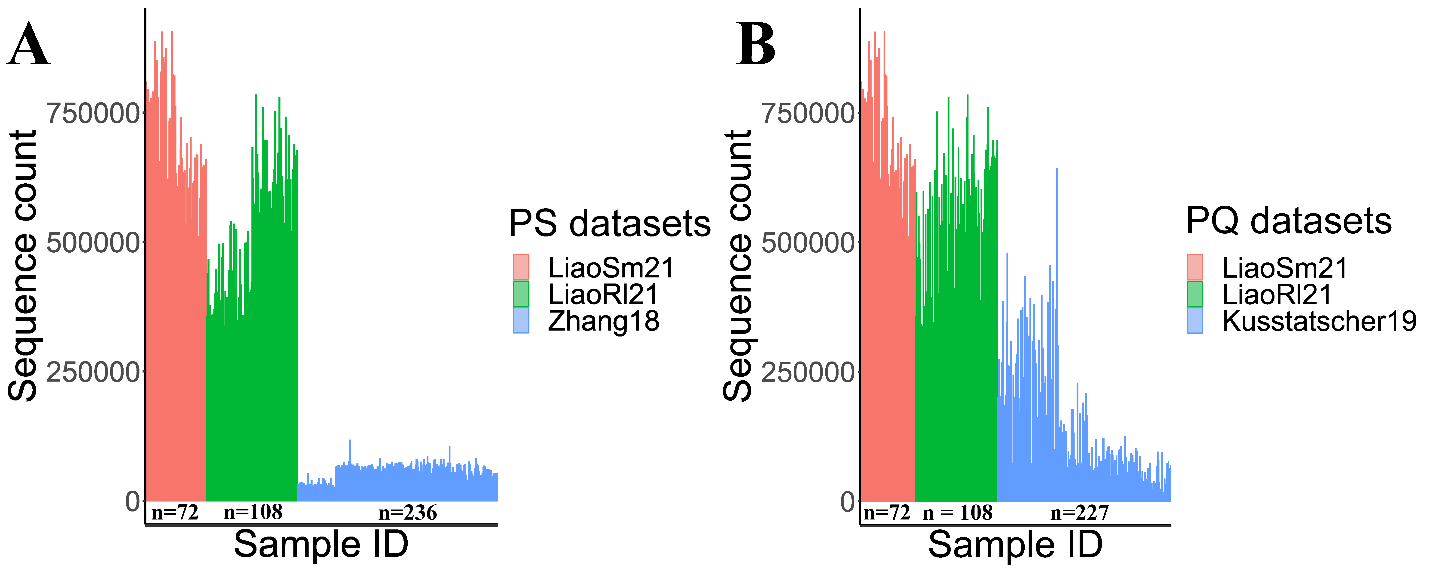


**Figure S1.** The 16S rRNA gene sequencing depth for microbiota samples from produce safety (PS) datasets and produce quality (PQ) datasets. (A) Distribution of 16S rRNA gene amplicon sequence count across the three PS datasets, including LiaoSm21, LiaoRl21, and Zhang18. (B) Distribution of 16S rRNA gene amplicon sequence count across the three PQ datasets, including LiaoSm21, LiaoRl21, and Kusstatscher19.


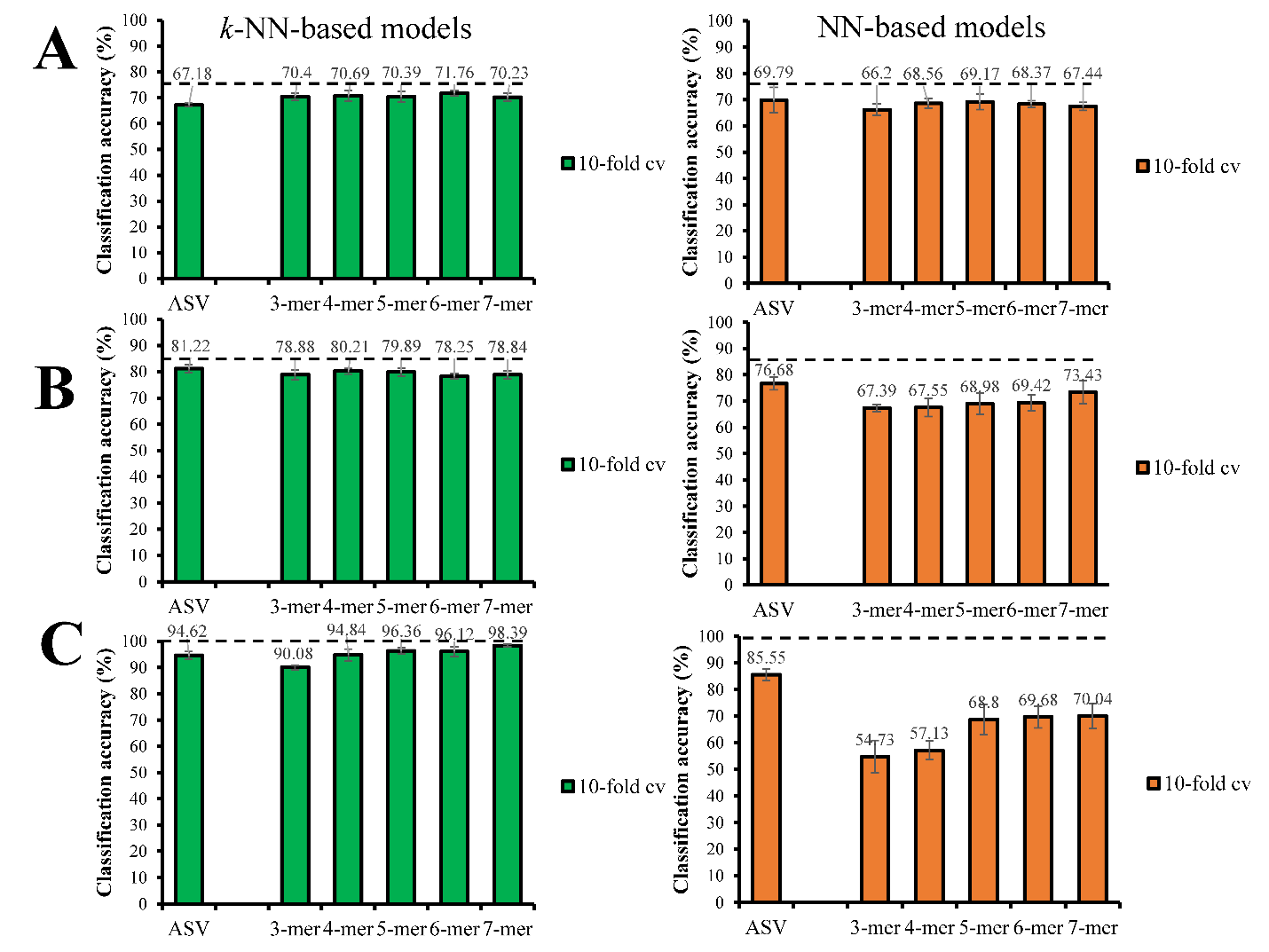


Figure S2. Comparison of the classification performance of PS classifiers based on k-NN and NN by using ASV datasets and hash datasets from 3-mer to 7-mer. (A) Accuracy of PS classifiers based on k-NN and NN using ASV and 3-mer to 7-mer hash datasets of Zhang18. The trained models were validated by using the 10-fold CV method. (B) Accuracy of PS classifiers based on k-NN and NN using ASV and 3-mer to 7-mer hash datasets of LiaoRl21. (C) Accuracy of PS classifiers based on k-NN and NN using ASV and 3-mer to 7-mer hash datasets of LiaoSm21. The black dash lines represent the PS classification accuracy of RF-based models using 7-mer hash datasets.


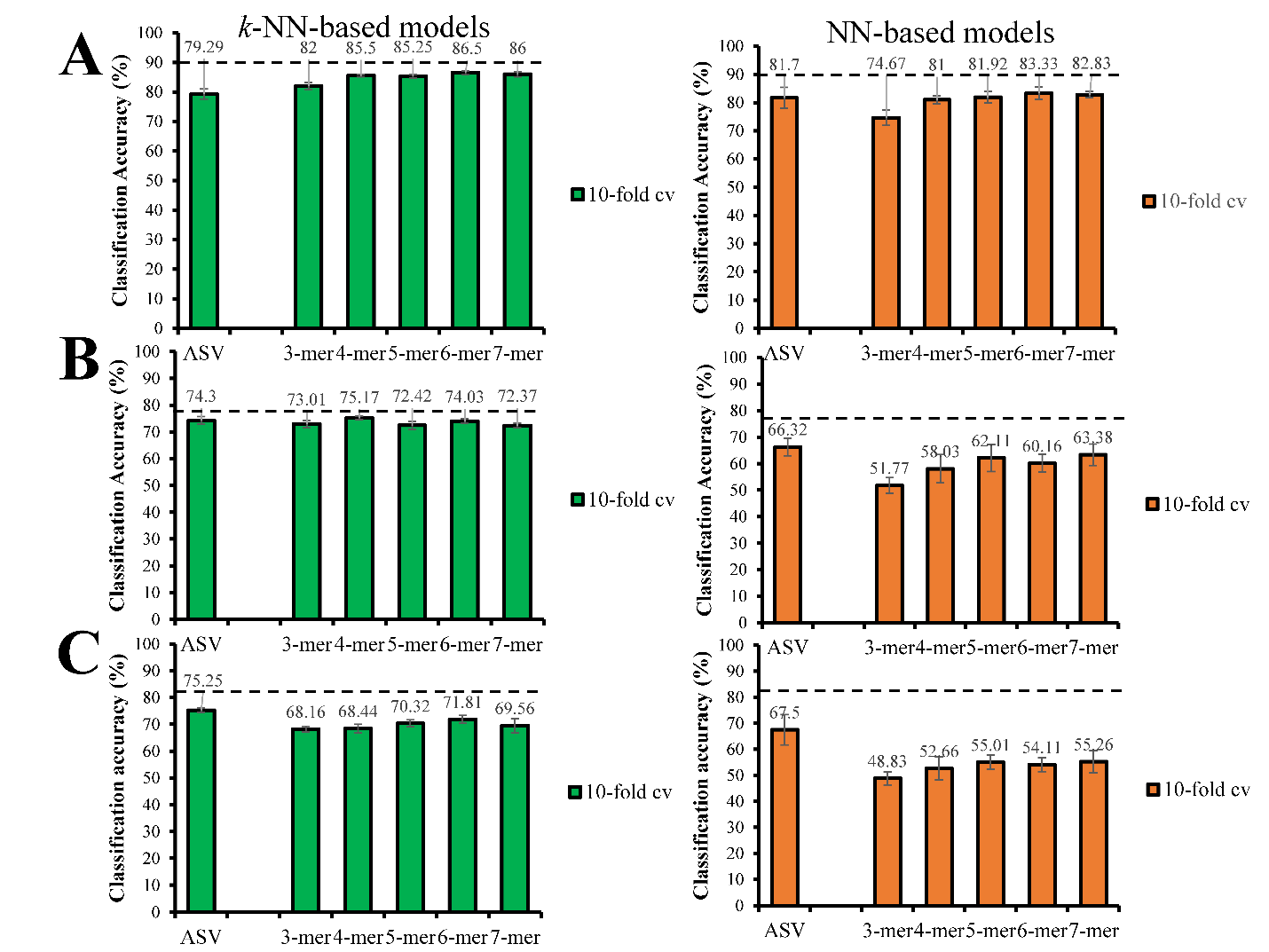


Figure S3. Comparison of the classification performance of PQ classifiers based on k-NN and NN by using ASV datasets and hash datasets from 3-mer to 7-mer. (A) Accuracy of PQ classifiers based on k-NN and NN using ASV and 3-mer to 7-mer hash datasets of Kusstatcher19 project. The trained models were validated by using the 10-fold CV method. (B) Accuracy of PQ classifiers based on k-NN and NN using ASV and 3-mer to 7-mer hash datasets of LiaoRl21 project. (C) Accuracy of PQ classifiers based on k-NN and NN using ASV and 3-mer to 7-mer hash datasets of LiaoSm21 project. The black dash lines represent the PQ classification accuracy of RF-based models using 7-mer hash datasets.


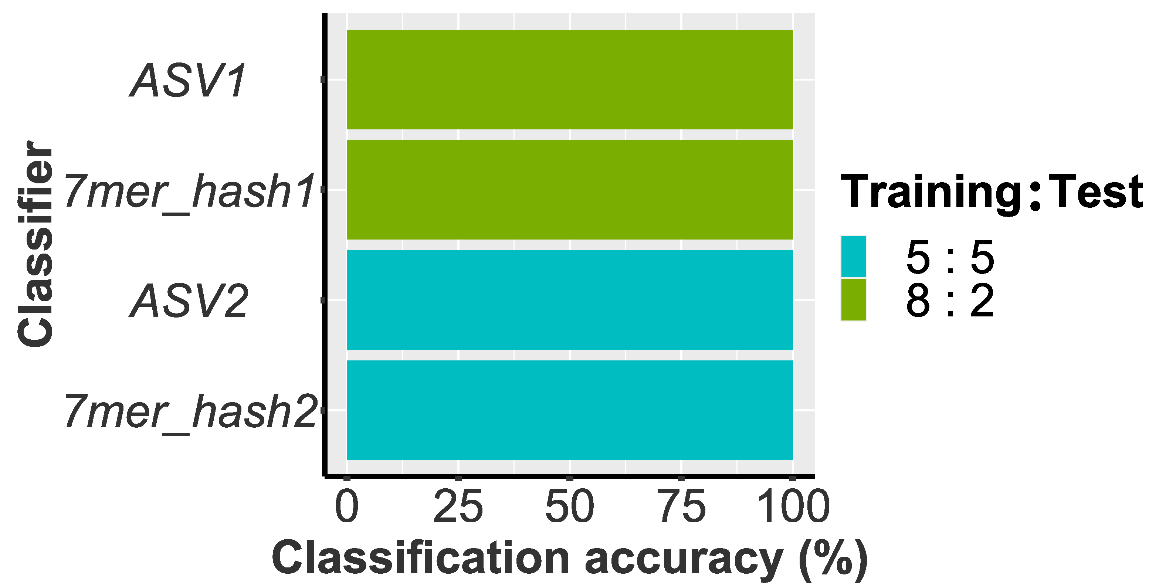


**Figure S4.** Classification performance of RF-based models established using the LiaoSm21 dataset of ASV and 7-mer hash representations in a ratio of training set to test set as 5:5 and 8:2, respectively.


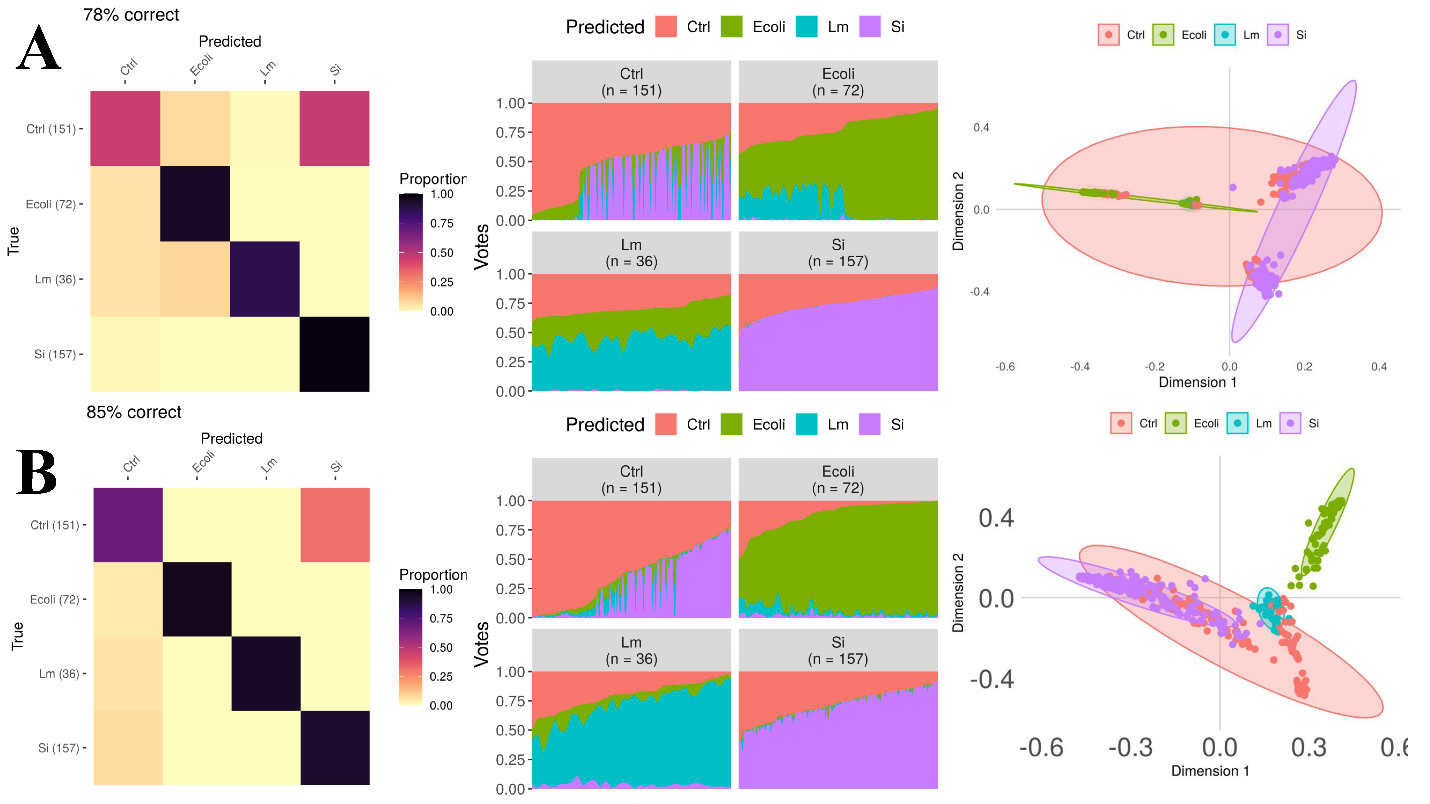


Figure S5. Plots of the confusion matrix (left), classification votes (middle), and MDS (right) generated from the RF-based models by using integrated ASV and 7-mer hash datasets associated with PS based on pathogen labels. (A) RF-based models established by using integrated ASV datasets. (B) RF-based models established by using integrated 7-mer hash datasets. The circles with color around the sample dots on MDS plots indicate the classification to Ctrl: control samples (red), Ecoli: *E. coli* O157:H7 contaminated samples (green), Lm: *L. monocytogenes* contaminated samples, and Si: *Salmonella* Infantis contaminated samples (purple).


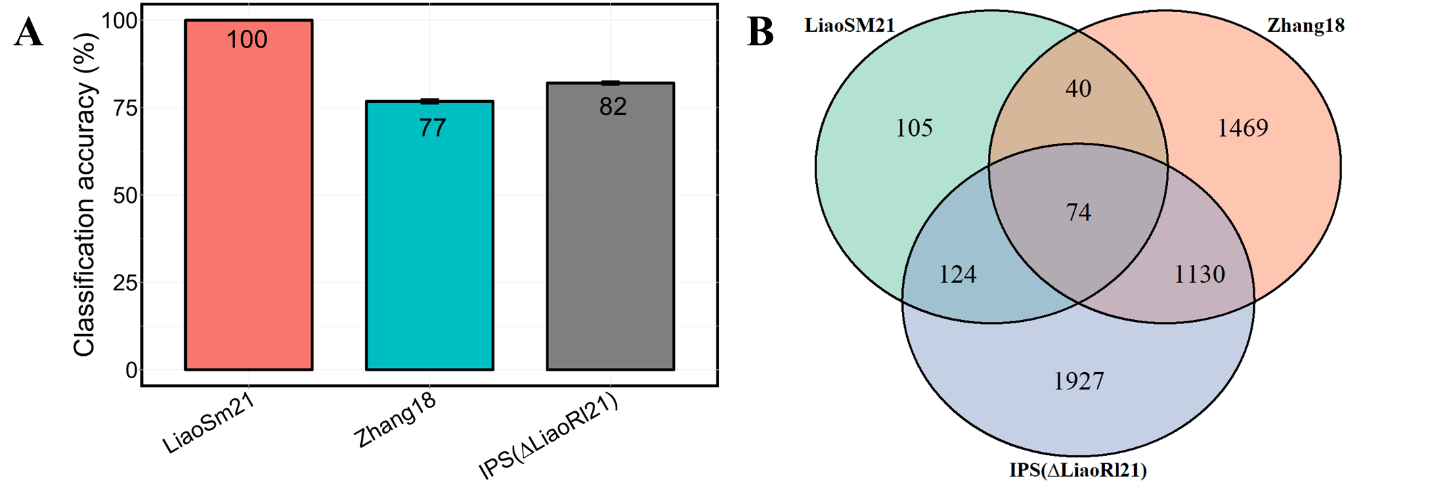


**Figure S6.** Comparison of training produce safety classification accuracy of classifiers trained on three training sets and a summary of the different and common features among them. (A) Classifiers were trained on the individual LiaoSm21 or Zhang18 7-mer hash dataset, and a classifier was trained on an integrated 7-mer hash dataset excluding LiaoRl21, denoting IPS (∆LiaoRl21). (B) The different and shared 7-mer hash features with positive mean decrease in accuracy (MDA) from the three classifiers were summarized.


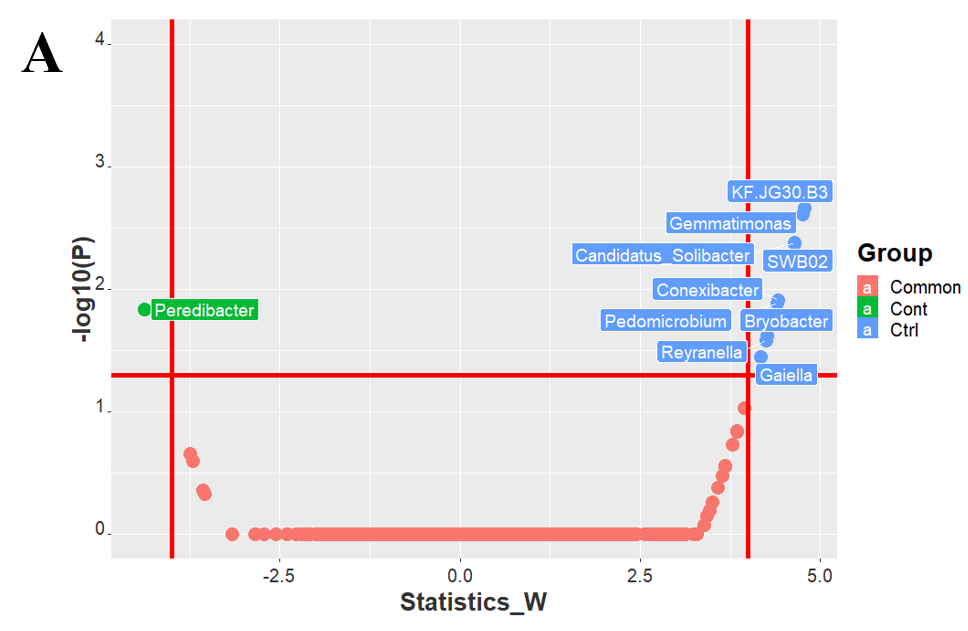


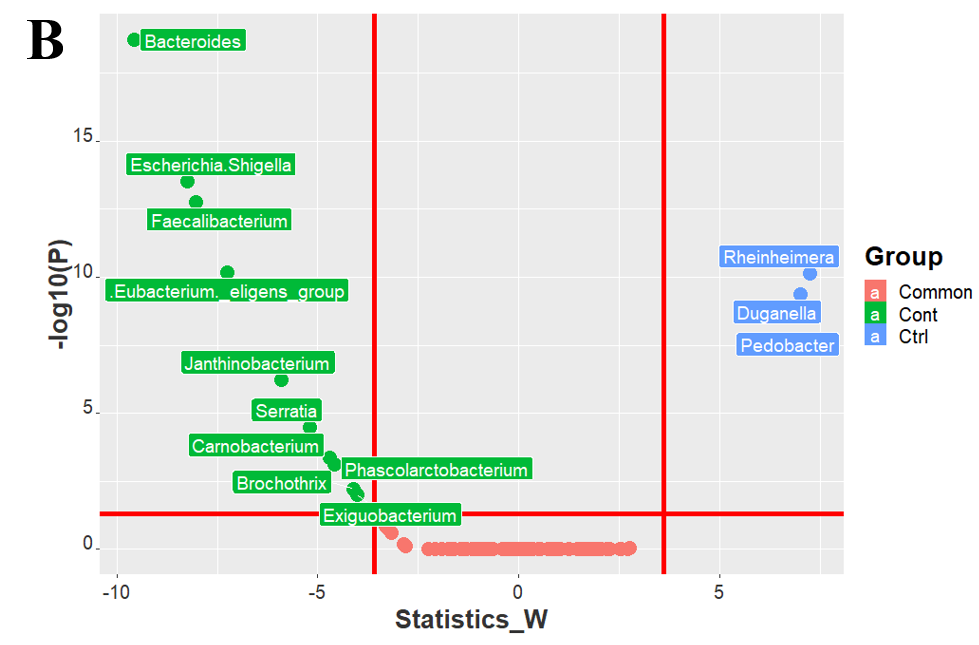


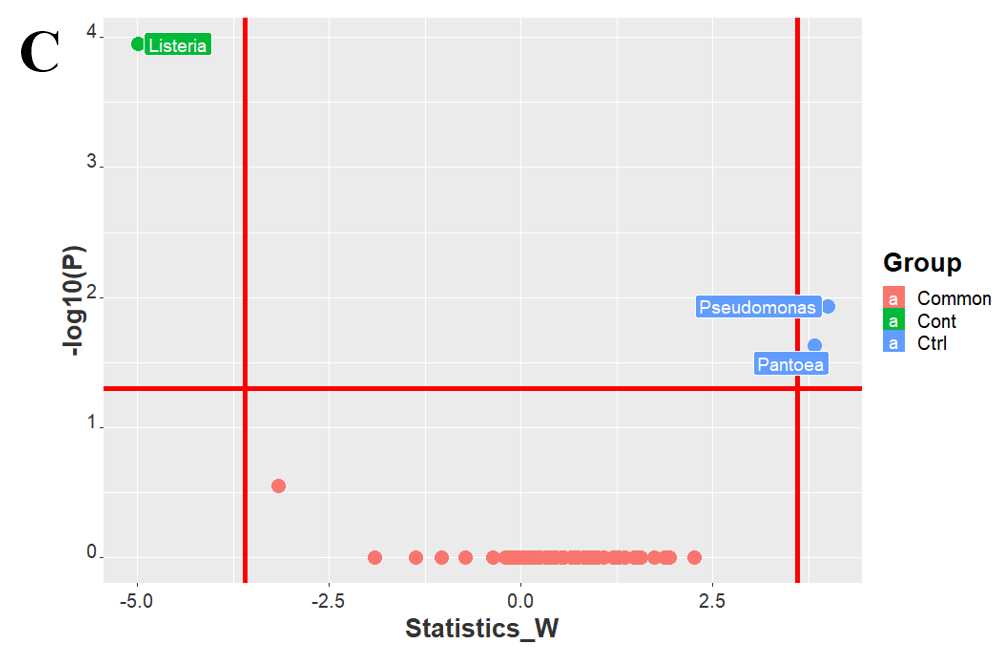


Figure S7. Taxonomic differential abundance analysis of individual fresh produce microbiome dataset related to PS. (A) Volcano plot of the significantly differential abundance of identified bacteria at the genus level in different groups of samples from Zhang18. (B) Volcano plot of the significantly differential abundance of identified bacteria at the genus level in different groups of samples from LiaoSm21. (C) Volcano plot of significantly differential abundance of identified bacteria at the genus level in samples from LiaoRl21. Common represents bacteria with no significantly different abundance between non-contamination group (Ctrl) and contamination group (Cont).


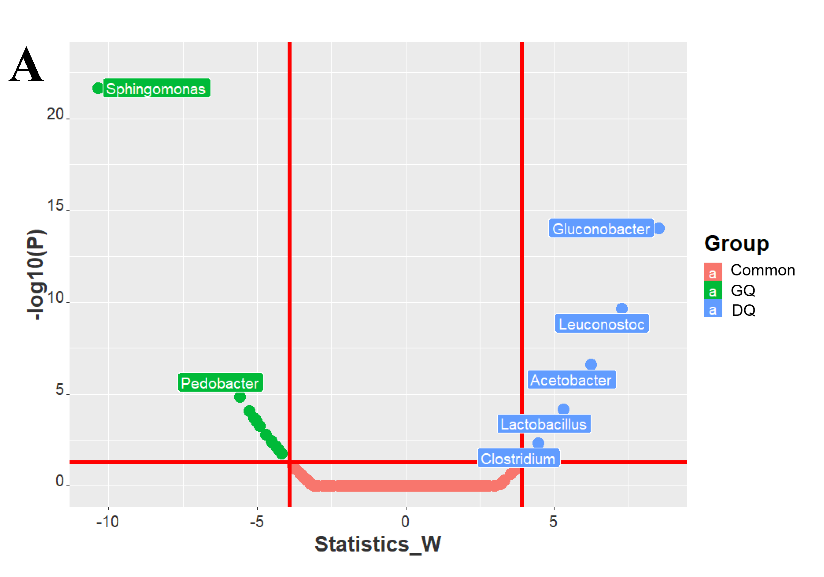


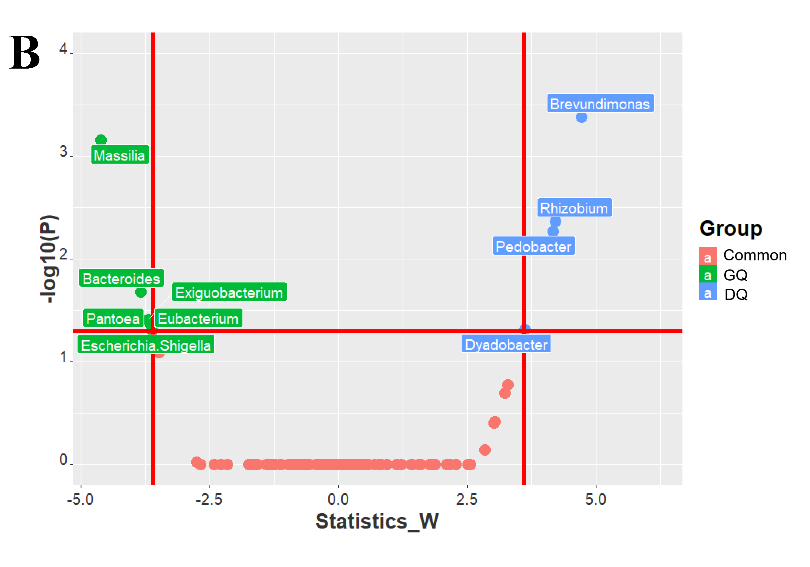


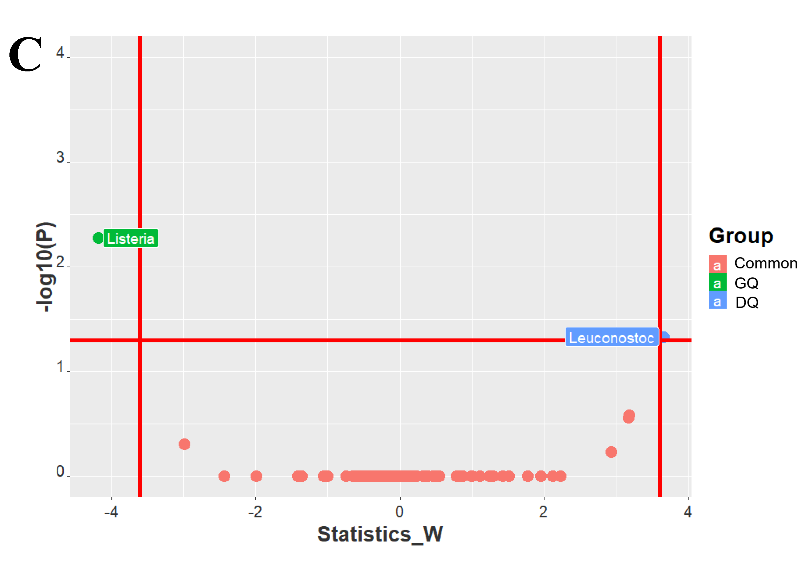


Figure S8. Taxonomic differential abundance analysis of individual fresh produce microbiome dataset related to PQ. (A) Volcano plot of significantly differential abundance of identified bacteria at the genus level in different group of samples from Kusstatscher19. (B) Volcano plot of significantly differential abundance of identified bacteria at the genus level in different groups of samples from LiaoSm21. (C) Volcano plot of significantly differential abundance of identified bacteria at the genus level in samples from LiaoRl21. Common represents bacteria with no significantly different abundance between good-quality group (GQ) and decreasing-quality group (DQ).


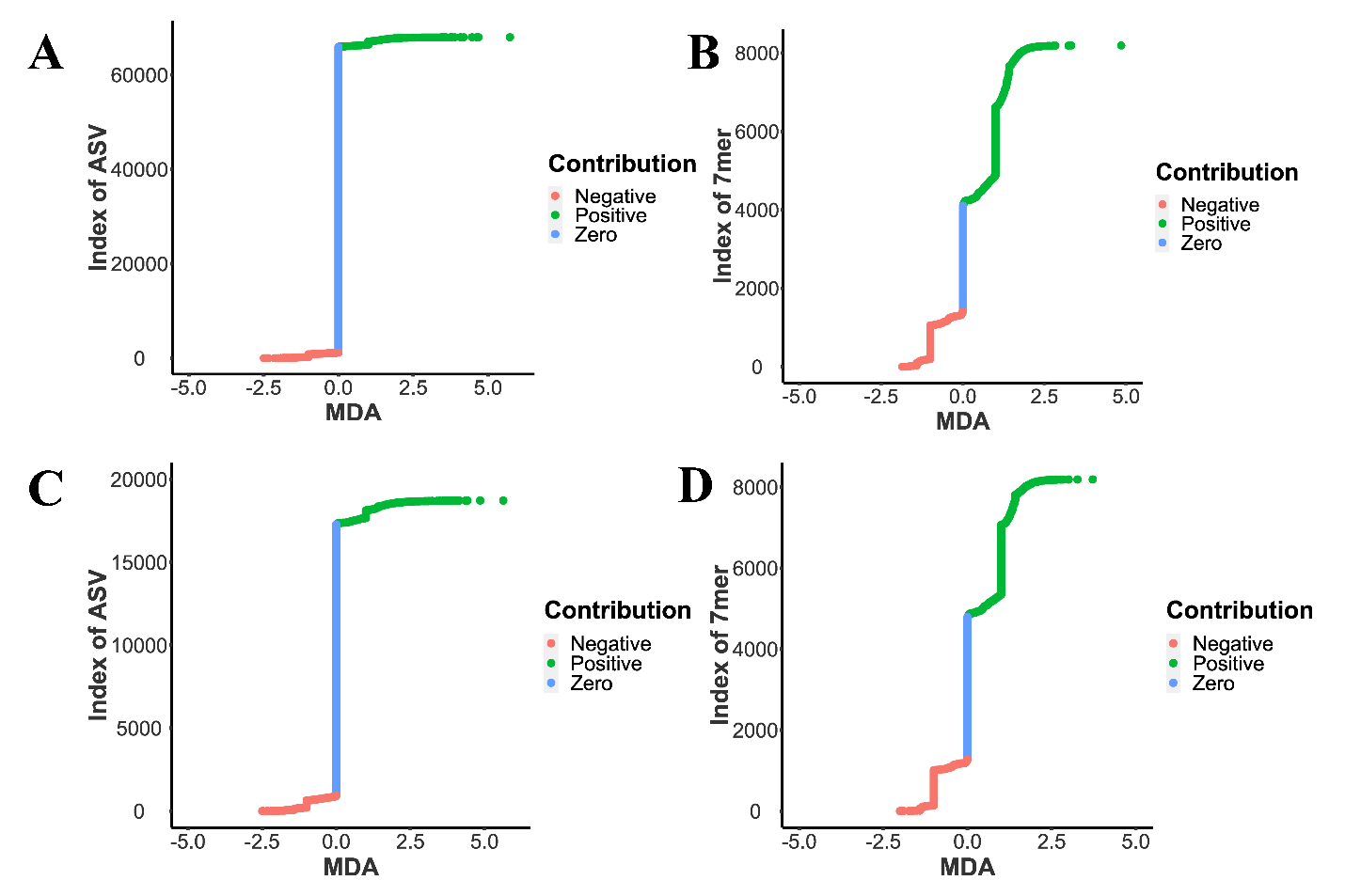


**Figure S9.**  Importance of features evaluated by mean decrease accuracy (MDA) provided by random forests-based classifiers. (**A**) Contribution of ASV features to PS classification identified from RF-based IPS classifiers. (**B**) Contribution of 7-mer hash features to PS classification identified from RF-based IPS classifiers. (**C**) Same as **(A)**, but for PQ. (**D**) Same as **(B)**, but for PQ.


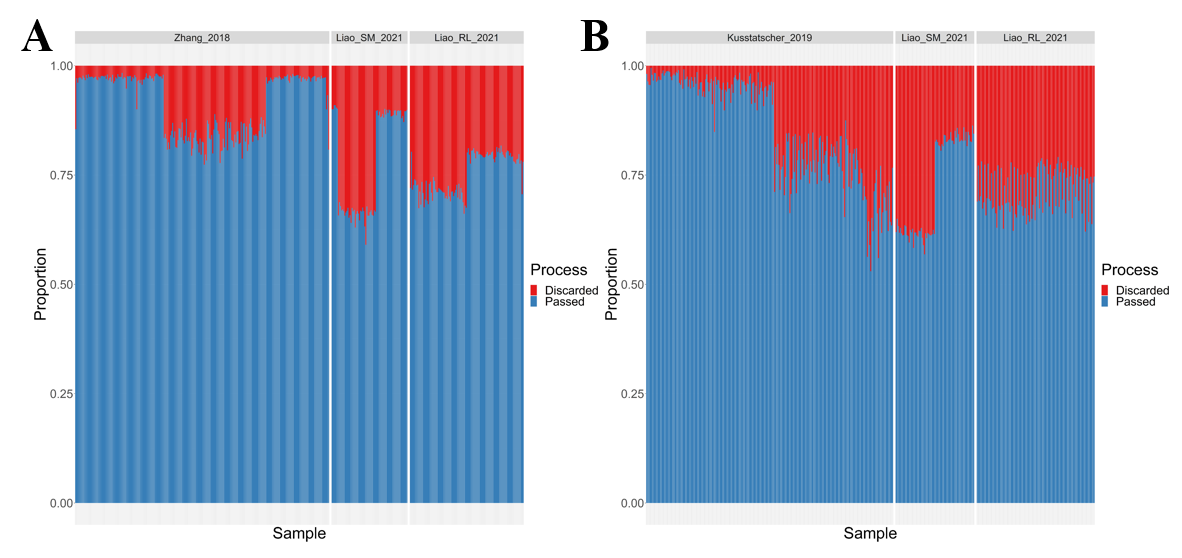


Figure S10. The proportion of sequence discarded from 16S rRNA sequence datasets of fresh produce microbiota related to PS and PQ during the denoising step by using the DADA2 plugin in QIIME 2. (A) Sequence loss from three microbiome sequence datasets associated with PS, including projects Zhang18, LiaoSm21, and LiaoRl21. (B) sequence loss from three microbiome sequence datasets associated with PQ, including projects Kusstatscher19, LiaoSm21, and LiaoRl21.


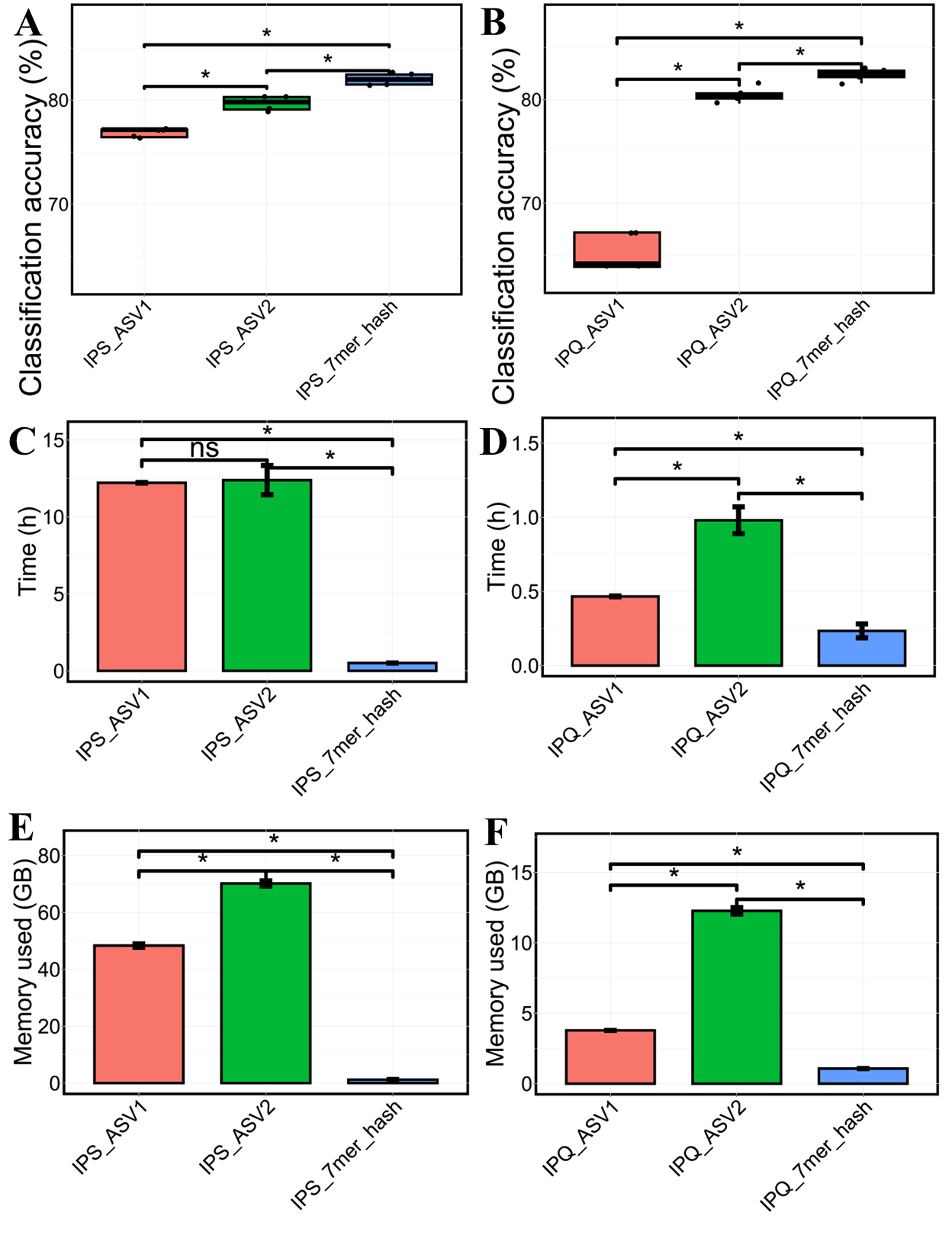


**Figure S11.** Comparison of produce safety (PS) and produce quality (PQ) classification performance between RF-based models constructed using denoised ASV datasets, un-denoised ASV datasets, and 7-mer hash datasets. (A) and (B), PS and PQ classification accuracy of models using denoised ASV datasets, un-denoised ASV datasets, and 7-mer hash datasets, respectively. (C) and (D), computing time (h) of PS and PQ classification by the models using the three types of datasets mentioned above. (E) and (F), computing memory use (GB) of PS and PQ classification by the models. The “*” strands for *P* < 0.05, indicating significant differences present between groups of samples determined by the non-parametric Wilcoxon rank sum test. IPS: integrated PS; IPQ: integrated PQ; h: hour; GB: Gigabyte.
