# Supplementary material for: Microbiome-based classification models for fresh produce safety and quality evaluation
Source: Microbiol Spectr. 2024 Mar 6;12(4):e03448-23. doi: 10.1128/spectrum.03448-23 (PMC10986475; doi:10.1128/spectrum.03448-23)
Supplement: Supplemental tables — Tables S1 to S5. [file spectrum.03448-23-s0002.docx]

Table S1. The number of case and control samples in each fresh produce microbiome dataset.

| Dataset | Class | Total sample number | Case number (Cont/DQ) | Control number (Ctrl/GQ) |
| --- | --- | --- | --- | --- |
| Zhang18 | PS | 236 | 158 | 78 |
| LiaoSm21 |  | 72 | 36 | 36 |
| LiaoRl21 |  | 108 | 72 | 36 |
| Kusstatscher19 | PQ | 227 | 156 | 71 |
| LiaoSm21 |  | 72 | 45 | 27 |
| LiaoRl21 |  | 108 | 54 | 54 |

PS and PQ represent produce safety and produce quality; Cont and Ctrl stand for contaminated group and non-contaminated group; GQ and DQ mean good-quality group and decreasing-quality group.

**Table S2.** The top 10 most important unique features based on MDA related to the PS in LiaoSm21 and Zhang18 7-mer hash datasets compared to the IPS (∆LiaoRl21) 7-mer hash dataset.

| 7-mer hash dataset | Hash index | MinHash ID | MDA |
| --- | --- | --- | --- |
| LiaoSm21 | H3102 | 6927575583435150000 | 0.0037 |
|  | H6818 | 15285158015323200000 | 0.0034 |
|  | H3416 | 7667092637882800000 | 0.0033 |
|  | H2371 | 5220445030488060000 | 0.0030 |
|  | H868 | 1907607021641350000 | 0.0030 |
|  | H3008 | 6738411152408340000 | 0.0030 |
|  | H2045 | 4485876426544520000 | 0.0029 |
|  | H2013 | 4442128602610340000 | 0.0028 |
|  | H5114 | 11435248343217600000 | 0.0028 |
|  | H2008 | 4436399599970260000 | 0.0027 |
| Zhang18 | H5207 | 11611399149064000000 | 0.0011 |
|  | H7798 | 17580864743247400000 | 0.00057 |
|  | H5634 | 12588975449885600000 | 0.00057 |
|  | H406 | 882600614311160000 | 0.00053 |
|  | H5989 | 13395678743523300000 | 0.00052 |
|  | H6889 | 15445042116747300000 | 0.00049 |
|  | H7354 | 16587073802930700000 | 0.00045 |
|  | H4803 | 10752721176425800000 | 0.00044 |
|  | H5733 | 12831836189399800000 | 0.00042 |
|  | H7271 | 16352664567049800000 | 0.00041 |

*MDA: Mean decrease accuracy; IPS: Integrated produce safety.

Table S3. *P* values of the indicators identified by the ANCOM-BC analysis for fresh produce in different contamination and pathogen groups.

| Indicator | Group | *P* value |
| --- | --- | --- |
| *Rheinheimera* | Ctrl (C) | 1.19 × 10^-5^ |
| *Pseudomonas* | Ctrl (C) | 8.41 × 10^-3^ |
| *Escherichia-Shigella* | Cont | 1.98 × 10^-4^ |
| *Listeria* | Cont | 1.53 × 10^-3^ |
| *Bacteroides* | Cont | 1.57 × 10^-3^ |
| *Peredibacter* | Cont | 6.69 × 10^-3^ |
| *Faecalibacterium* | Cont | 0.048 |
| *Rheinheimera* | Ctrl (P) | 3.81 × 10^-7^ |
| *Pedobacter* | Ctrl (P) | 1.51 × 10^-5^ |
| *Duganella* | Ctrl (P) | 5.15 × 10^-4^ |
| *Pseudomonas* | Ctrl (P) | 5.16 × 10^-3^ |
| *Listeria* | *L. moncytogenes* | 1.39 × 10^-10^ |
| *Escherichia-Shigella* | *E. coli* O157:H7 | 6.97 × 10^-10^ |
| *Faecalibacterium* | *E. coli* O157:H7 | 3.56 × 10^-6^ |
| *Bacteroides* | *E. coli* O157:H7 | 4.34 × 10^-5^ |
| *Eubacterium* | *E. coli* O157:H7 | 2.57 × 10^-3^ |
| *Ruminococcus* | *E. coli* O157:H7 | 0.019 |
| *Sanguibacter* | *E. coli* O157:H7 | 0.032 |
| *Peredibacter* | *Salmonella* Infantis | 3.37 × 10^-3^ |

Ctrl (C) represents the non-contaminated samples under the contamination groups; Ctrl (P) stands for the non-pathogenic samples under the pathogen groups.

Table S4. *P* values of the indicators identified by the ANCOM-BC analysis for fresh produce in different quality groups.

| Indicator | Group | *P* value |
| --- | --- | --- |
| *Sphigomonas* | GQ | 4.54 × 10^-9^ |
| *Pedobacter* | GQ | 3.16 × 10^-4^ |
| *Parablastomonas* | GQ | 2.31 × 10^-3^ |
| *Paracoccus* | GQ | 7.09 × 10^-3^ |
| *Pir4_lineage* | GQ | 0.010 |
| *Nocardioides* | GQ | 0.048 |
| *Leuconostoc* | DQ | 8.23 × 10^-9^ |
| *Gluconobacter* | DQ | 1.38 × 10^-7^ |
| *Lactobacillus* | DQ | 1.16 × 10^-4^ |
| *Acetobacter* | DQ | 5.8 × 10^-4^ |
| *Clostridium* | DQ | 0.015 |

GQ represents good quality; DQ means decreasing quality.

Table S5. The number of features with a positive or negative contribution to the PS and PQ classification based on mean decrease accuracy provided by RF-based models.

| Dataset | Class | Total number of ASV | Number of positive features (ASV) | Number of negative features (ASV) | Total number of 7-mer hash | Number of positive features (7-mer hash) | Number of negative features (7-mer hash) |
| --- | --- | --- | --- | --- | --- | --- | --- |
| Zhang18 | PS | 49404 | 2029 | 1253 | 8192 | 2665 | 1506 |
| LiaoSm21 |  | 2510 | 401 | 46 | 8192 | 349 | 0 |
| LiaoRl21 |  | 2365 | 228 | 276 | 8192 | 1114 | 543 |
| IPS |  | 68007 | 2057 | 1179 | 8192 | 4084 | 1408 |
| Kusstatscher19 | PQ | 1164 | 210 | 66 | 8192 | 2070 | 603 |
| LiaoSm21 |  | 2510 | 354 | 222 | 8192 | 908 | 448 |
| LiaoRl21 |  | 2365 | 278 | 238 | 8192 | 1469 | 654 |
| IPQ |  | 18732 | 1451 | 918 | 8192 | 3403 | 1271 |

PS and PQ represent produce safety and produce quality; IPS and IPQ stand for integrated produce safety and integrated produce quality. ASV means amplicon sequence variant.

**Materials and methods**

**1. Romaine lettuce salad**

A total of 72 bagged commercial RL products were purchased from local grocery stores during the early harvest season and the late harvest season (1, 2), including three brands (A, 255g/bag; B, 198 g/bag; and C, 284 g/bag) with two brands (A and B) from California and one brand (C) from Florida. Once arrived at the laboratory, the samples were stored at 4^o^C and were sampled on their labeled “use-by” dates (Day 0) and 5 days after the “use-by” dates (Day 5) for evaluation of the microbial communities present in these products.

**2. Inoculation of Romaine lettuce**

A 3-strain *Escherichia coli* O157:H7 (ECO157) cocktail was prepared for inoculating RL. These three strains included AU 301 and 305 (two ECO157 strains obtained from Auburn University) and ECO157 505 B, a beef outbreak strain. The 3-strain *Listeria monocytogenes* (LM) composed of LM 101M (serotype 4b, a beef and pork sausage isolate), LM 108M (serotype 1/2b, a hard salami isolate), and 10403S (serotype 1/2a, a strain obtained from the UC Davis Glenn Young lab). To activate the culture, frozen culture stocks from -80°C freezer were thawed on ice followed by streaking a loop of thawed cultures onto TSA agar and incubated at 37°C for 24 hours. After that, a single colony was picked from each plate and transferred into 10 ml of fresh TSB and incubated at 37°C for 24 hours. A loop of fresh broth culture was transferred again into 10 ml of fresh TSB and incubated at 37°C overnight (18 hours) before use. Each fresh overnight culture was washed with 10 ml of phosphate buffered solution (PBS, pH = 7.4) twice via centrifugation. The OD_600_ of each washed culture was adjusted to the value of 1.60 by using PBS solution via an Ultrospec 10 Cell Density Meter (Amrsham BioSciences, Piscataway, NJ). To prepare the cocktail, 1 ml of each washed pathogen culture was mixed to form the 3-strain cocktail. The final concentrations were 9.03 ± 0.64 Log CFU/ml and 9.49 ± 0.58 Log CFU/ml for ECO157 and LM cocktails respectively. One milliliter of each cocktail was injected into each commercial bag of RL by using a BD 1 ml syringe with a 26 G × 3/8” detachable needle (Becton Dickinson and Company, Franklin Lakes, NJ). The penetration hole on the bag was immediately sealed with tape to maintain the same package atmosphere. After inoculation, the inoculated bag was vigorously shaken for 1 min to distribute the inoculum evenly (3). RL samples were inoculated on the “use-by” dates (Day 0) during storage. The inoculated samples were then stored at 4°C and analyzed after 5 days of storage.

**3. Inoculated Romaine lettuce processing**

For each season, 18 ECO157 inoculated bagged salad (ECRL) and 18 LM inoculated bagged salad LMRL were sampled on Day 0 and Day 5. To homogenate each salad sample, 80 grams of chopped RL were homogenized by using the SmasherTM Lab Blender (AES-Chemunix, Bruz, France) for 120 s at the fast speed (620 strokes/min) with 320 ml of phosphate-buffered saline (PBS, pH 7.4) in a 55 oz Whirl-Pak filter bag (Nasco, Fort Atkinson, WI, USA). Ten milliliters of each sample homogenate were centrifuged for collecting pellets and were then used for DNA extraction.

**4. DNA extraction**

The inoculated homogenate pellets were washed twice by using 10 ml of PBS via centrifugation, re-suspended with 1 mL of PBS and transferred into 1.5 mL micro-centrifuge tubes (VWR, Atlanta, GA). For each season, DNA from 18 uninoculated Romaine lettuce samples (RL), 18 ECO157 inoculated RL samples (ECRL), and 18 LM inoculated RL samples (LMRL) were extracted using the DNeasy Powersoil kit (Qiagen, Gaithersburg, MD) following the manufacturer’s instructions. A total of 108 DNA samples were extracted and stored at -80 °C for 16S rRNA gene sequencing analyses.

**5. 16S rRNA gene sequencing**

The library construction was built based on the amplification of V3-V4 region of 16S rRNA gene (341F: 5’-CCTACGGGNGGCWGCAG-3’ and 785R: 5’-GACTACHVGGGTATCTAATCC-3’). The amplicons were tagged with 7-bp forward sample-specific barcodes (4). The sequencing was performed by using Illumina^®^ MiSeq instrument with using MiSeq Reagent Kit v3 (Illumina, CA) to produce 2 × 300 bp paired-end reads. The library preparation and the sequencing were conducted at the Hudsonalpha Genomic Service Laboratory (Huntsville, AL). The de-multiplexed sequences were obtained from Illumina BaseSpace platform by assigning reads to each sample based on the sample-specific barcodes.

**References**

1. Liao C, Wang L. 2021. Evaluation of the bacterial populations present in Spring Mix salad and their impact on the behavior of *Escherichia coli* O157:H7. Food Control 107865.

2. Williams TR, Moyne A-L, Harris LJ, Marco ML. 2013. Season, irrigation, leaf age, and *Escherichia coli* inoculation influence the bacterial diversity in the lettuce phyllosphere. PLoS One 8:e68642.

3. Zeng W, Vorst K, Brown W, Marks BP, Jeong S, Pérez-Rodríguez F, Ryser ET. 2014. Growth of *Escherichia coli* O157:H7 and *Listeria monocytogenes* in packaged fresh-cut romaine mix at fluctuating temperatures during commercial transport, retail storage, and display. J Food Prot 77:197–206.

4. Sinclair L, Osman OA, Bertilsson S, Eiler A. 2015. Microbial community composition and diversity via 16S rRNA gene amplicons: evaluating the illumina platform. PLoS One 10:e0116955.
